# Supplementary material for: Generalized contact matrices allow integrating socioeconomic variables into epidemic models
Source: Sci Adv. 2024 Oct 11;10(41):eadk4606. doi: 10.1126/sciadv.adk4606 (PMC11468902; doi:10.1126/sciadv.adk4606)
Supplement: 20241011-1 [file sciadv.adk4606.v1.pdf]

EPIDEMIOLOGY

Generalized contact matrices allow integrating socioeconomic variables into epidemic models

Adriana Manna[1], Lorenzo Dall'Amico[2], Michele Tizzoni[3], Márton Karsai[1,4], Nicola Perra[5]*

Copyright © 2024 The Authors, some rights reserved; exclusive licensee American Association for the Advancement of Science. No claim to original U.S. Government Works. Distributed under a Creative Commons Attribution License 4.0 (CC BY).

Variables related to socioeconomic status (SES), including income, ethnicity, and education, shape contact structures and affect the spread of infectious diseases. However, these factors are often overlooked in epidemic models, which typically stratify social contacts by age and interaction contexts. Here, we introduce and study generalized contact matrices that stratify contacts across multiple dimensions. We demonstrate a lower-bound theorem proving that disregarding additional dimensions, besides age and context, might lead to an underestimation of the basic reproductive number. By using SES variables in both synthetic and empirical data, we illustrate how generalized contact matrices enhance epidemic models, capturing variations in behaviors such as heterogeneous levels of adherence to nonpharmaceutical interventions among demographic groups. Moreover, we highlight the importance of integrating SES traits into epidemic models, as neglecting them might lead to substantial misrepresentation of epidemic outcomes and dynamics. Our research contributes to the efforts aiming at incorporating socioeconomic and other dimensions into epidemic modeling.

INTRODUCTION

Contact matrices have become an integral part of realistic epidemic models for respiratory infections. They encode interaction patterns through specific contact rates describing how frequently and for how long individuals belonging to different categories or groups meet each other. Age has become the most commonly used variable to define these groups. This way of stratification allows models that capture heterogeneous mixing rates observed among individuals of different ages (*1*–*6*). Young adults, for example, are usually very active and tend to interact more with other young adults (*7*, *8*). Elderly individuals, instead, tend to report the smallest number of contacts, and their role in the transmission dynamics of many respiratory infections is less relevant (*6*). Furthermore, distinguishing individuals according to age allows accounting for heterogeneities in transmission rates and epidemic outcomes. The observation that transmission rates are strongly influenced by age grabbed the attention of disease modelers decades ago (*9*), as they were investigating so-called childhood infections. Accordingly, the first studies to propose a deviation from the homogeneous mixing assumption addressed age-varying transmission rates given their practical importance for vaccination programs (*10*). More recently, age has also been identified as key in the case of COVID-19. Since the beginning of the pandemic, outcomes of COVID-19 infections were found to strongly depend on age, leading to a higher case fatality ratio in older populations (*11*).

Contact matrices can be stratified by considering other variables, beyond age. As the number and type of contacts considerably vary by location, the setting where interactions take place (i.e., household, workplace, school, and community) is another popular choice for stratification (*3*, *5*, *12*). Age and location are often considered jointly (*3*, *5*, *12*). Mixing patterns disaggregated by age and setting have been used to estimate age-specific and context-specific transmission parameters for epidemic models, guide public health policy, evaluate intervention strategies, and assess the risk of infection across population groups (*13*–*16*).

Despite their essential role, however, age and context are far from being the only important variables shaping contact patterns, disease dynamics, and epidemic outcomes. Individual characteristics related to socioeconomic status (SES) such as wealth, race, ethnicity, occupation, and education, among others, have been shown to play a key role in the transmission of infectious diseases (*17*–*20*). From the influenza pandemics of 1918 and 2009 (*21*, *22*) to the West African Ebola outbreak (*23*) and the COVID-19 pandemic (*20*, *24*–*34*) individuals experiencing a lower SES have been consistently associated with higher rates of infections, deaths, as well as reduced access to care and ability to comply with nonpharmaceutical interventions (NPIs).

Despite the recognized importance of SES in the transmission dynamics of close-contact infections, the overwhelming majority of epidemic models neglect these dimensions (*17*–*20*). SES is often considered only a posteriori in analyses of models' outputs (e.g., number of deaths or cases) but rarely enters the core of their formulation as age and location do. The roots of this shortcoming can be traced back to the lack of empirical surveys of social contact data including characteristics of the respondents and their peers to account for SES (*7*, *35*). The lack of data has also hampered the development and exploration of general modeling frameworks designed to include these features in their core (*20*).

Here, we present an epidemic framework featuring generalized contact matrices stratified by any number of dimensions (i.e., variables). As a first step, we study the mathematical properties of such a framework by considering age plus m other, unspecified, dimensions. We focus on prototypical susceptible-exposed-infectious-recovered (SEIR) compartmental models and derive a closed-form expression for the basic reproductive number R_0. The derivation follows the next-generation matrix approach (*36*, *37*) applied to a flattened representation of generalized contact matrices. We quantify how much R_0 differs from the correspondent value computed in models including only one attribute (e.g., age). We prove a lower-bound theorem showing how increasing the number of dimensions in contact matrices

[1]Department of Network and Data Science, Central European University, Vienna, Austria. [2]ISI Foundation, Turin, Italy. [3]Department of Sociology and Social Research, University of Trento, Trento, Italy. [4]National Laboratory for Health Security, HUN-REN Rényi Institute of Mathematics, Budapest, Hungary. [5]School of Mathematical Sciences, Queen Mary University of London, London, UK.
*Corresponding author. Email: n.perra@qmul.ac.uk

cannot decrease R_0. Furthermore, we prove that building generalized contact matrices via a random mixing assumption for the additional dimensions (i.e., contact rates are set proportional to the respective subgroup sizes) leads to the same R_0 of lower dimensional contact representations. Conversely, we numerically observe that correlations in contact patterns, such as mixing assortativity and heterogeneous activity, increase the value of R_0. In the second step of our analysis, we highlight the importance of integrating SES dimensions into epidemic models and showcase how the proposed framework opens avenues in this direction. Starting from synthetic scenarios, we show how adopting generalized contact matrices allows quantifying heterogeneities in epidemic outcomes and estimating the impact of NPIs across socioeconomic strata. It enables accounting not only for differences in contact patterns but also for heterogeneities in adherence to NPIs across subgroups of the population. We then apply our framework to empirical data from Hungary. We quantify the possible misrepresentation induced by neglecting SES in simulated outbreaks. The results confirm notable differences between models. The use of generalized contact matrices, which stratify contacts by age and one SES dimension, in agreement with the mathematical derivations, leads to higher values of R_0 for a given disease, to lower values of attack rates for a given R_0, and allows capturing heterogeneity in disease's burden across SES groups. Overall, the proposed framework allows for more expressive epidemic models that can capture a broader range of key, and otherwise transparent, dynamics.

RESULTS

We consider a SEIR compartmental model where susceptible (S) are healthy individuals at risk of infection, exposed (E) are infected but not yet infectious, infectious (I) can spread the disease, and recovered (R) are no longer infectious nor susceptible to the disease (*38*). Within this setting, we propose a modeling framework that extends classic approaches by including generalized contact matrices.

Generalized contact matrices

We define generalized contact matrices as multidimensional objects that stratify contact patterns across any number of dimensions. Because age is often considered the key variable in traditional approaches, in what follows, we focus on age and other categorical dimensions. Hence, we move from the prototypical contact matrices $\mathbf{C}$, whose elements C_{ij} describe the contact rates between individuals in age brackets i and j, to $\mathbf{G}$, whose elements $G_{\mathbf{a},\mathbf{b}}$ capture the contact rates between individuals in subgroups $\mathbf{a}$ and $\mathbf{b}$. Here, $\mathbf{a} = (i, \alpha, \beta, \ldots, \gamma)$ and $\mathbf{b} = (j, \eta, \nu, \ldots, \xi)$ are index vectors (i.e., tuples) representing individual's membership to each category. We use Greek letters to highlight the difference between age and other dimensions, but the distinction among variables is not, in general, necessary. To provide a concrete example of a generalized contact matrix, we can imagine stratifying the population according to age ($i \in [1, \ldots, K]$), income ($\alpha \in [1, \ldots, V_1]$), and educational attainment ($\beta \in [1, \ldots, V_2]$). In this case, the generalized contact matrix $\mathbf{G}$ would describe the average number of contacts that an individual in age bracket i, income α, and education β has with individuals in age group j, income η, and education ν in a given time window (see Fig. 1). From this perspective, the matrix $\mathbf{C}$ can be thought of as an aggregation of contact rates at lower levels of stratification. As discussed in more detail in the Supplementary Materials, we can write $C_{ij} = N_i^{-1} \sum_{\mathbf{a}'\mathbf{b}'} G_{\mathbf{a}',\mathbf{b}'} N_{\mathbf{a}'}$. Here, $\mathbf{a}'$ and $\mathbf{b}'$ index all dimensions except age, N_i describes the number of individuals in age bracket i, and $N_{\mathbf{a}'}$ is the number of individuals in subgroup $\mathbf{a}'$. As with any aggregated metric, the elements of $\mathbf{C}$ are agnostic about the structure of contacts across the aggregated dimensions.

We denote with K the number of age groups, while with V_p ($p \in [1, \ldots, m]$) the number of subgroups in other dimensions. In this work, we use the words dimensions and variables as synonymous. To avoid confusion, we refer to the number of groups in each dimension as their size. While the generalized matrix $\mathbf{G}$ is a multidimensional object, the use of $T = K \prod_{p=1}^{m} V_p$ index vectors allows for a flattened representation in a squared two-dimensional (2D) matrix of size $T \times T$. The formulation can easily include different contexts where interactions take place, i.e., $G_{\mathbf{a},\mathbf{b}} = \sum_l \omega_l G_{\mathbf{a},\mathbf{b}}^{(l)}$, where ω_l captures the relative importance of each social setting in the transmission (*5*).

Epidemic model

As mentioned above, we study diseases whose natural history can be described by an SEIR compartmental model. Similar results can be

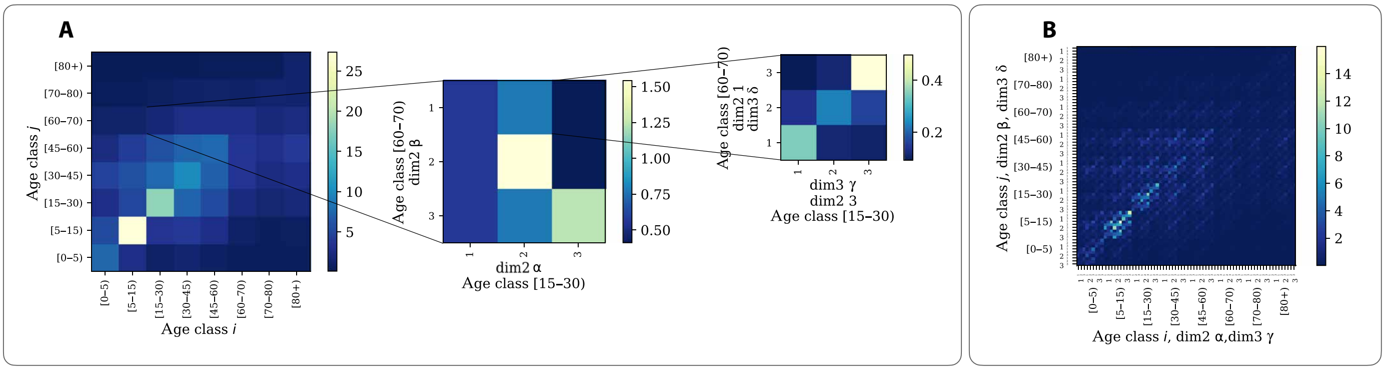

Fig. 1. Generalized contact matrices. (**A**) Schematic representation of generalized contact matrices. We can transform a $K \times K$ age-structured contact matrix $\mathbf{C}$ (first matrix on the left) into a generalized matrix $\mathbf{G}$ with $m + 1$ dimensions. Such transformation can be done according to different generalization schemes discussed in Materials and Methods and the Supplementary Materials. In the plot, we consider $m = 2$. Hence, the second matrix describes the stratification of contacts across a second dimension, i.e., $\alpha, \beta \in [1,2,3]$. The third matrix describes a further stratification, i.e., $\gamma, \delta \in [1,2,3]$. For simplicity, we show the stratification across the additional dimensions only for one entry of the lower level, respectively. (**B**) Example of a flattened generalized contact matrix including three dimensions $\mathbf{G}$ (72 × 72).

obtained for others such as the SIS and SIR models. To include the generalized contact matrices $\mathbf{G}$, we define $X_{\mathbf{a}}$ as the number of individuals in groups $\mathbf{a}$ and compartment X where $X \in [S, E, I, R]$. The model dynamics is described by the set of differential equations

$$\begin{aligned} d_t S_{\mathbf{a}}(t) &= -\Lambda_{\mathbf{a}}(t) S_{\mathbf{a}}(t) \\ d_t E_{\mathbf{a}}(t) &= \Lambda_{\mathbf{a}}(t) S_{\mathbf{a}}(t) - \Psi E_{\mathbf{a}}(t) \\ d_t I_{\mathbf{a}}(t) &= \Psi E_{\mathbf{a}}(t) - \Gamma I_{\mathbf{a}}(t) \\ d_t R_{\mathbf{a}}(t) &= \Gamma I_{\mathbf{a}}(t) \end{aligned} \tag{1}$$

where Γ is the recovery rate, and Ψ is the rate at which exposed individuals become infectious. The force of infection $\Lambda_{\mathbf{a}}(t) = \Phi \sum_{\mathbf{b}} G_{\mathbf{a},\mathbf{b}} \frac{I_{\mathbf{b}}(t)}{N_{\mathbf{b}}}$ describes the per capita rate at which susceptible individuals in categories $\mathbf{a}$ acquire the infection, and Φ is the transmissibility of the disease. For simplicity of exposition, we neglect demography (i.e., birth and death rates); hence, the size of the population is fixed. To avoid confusion with the indices of the additional dimensions, we denote the parameters regulating the disease dynamics with capital Greek letters.

Spectral properties of generalized contact matrices and the basic reproductive number

The basic reproductive number R_0 is one of the most important quantities of epidemiological relevance (*38*). It is defined as the number of secondary infections generated by a single infected seed in an otherwise susceptible population. The R_0 of an SEIR model, featuring a single-attribute contact matrix $\mathbf{C}$ defined, for example, by K age brackets, can be obtained using the next-generation matrix approach [see (*36*, *37*) for overviews of this method]. The expression reads $R_0 = \frac{\Phi}{\Gamma}\rho(\tilde{\mathbf{C}})$, where ρ denotes the spectral radius of the matrix $\tilde{\mathbf{C}} \in \mathbb{R}^{K\times K}$ whose elements are $\tilde{C}_{ij} = \frac{C_{ij}N_i}{N_j}$ (see the Supplementary Materials for the full derivation). As mentioned above, N_i and N_j describe the number of individuals in age brackets i and j, respectively. We can write $\tilde{\mathbf{C}} = \mathbf{R}\mathbf{N}^{-1}$, where $\mathbf{N}$ is a diagonal matrix whose elements are the number of individuals in each age group, and $\mathbf{R} = \mathbf{N}\mathbf{C}$ is a symmetric matrix describing the total number of contacts for each pair of age groups. How does this expression change when we consider generalized contact matrices? As shown in detail in the Supplementary Materials, the next-generation matrix approach can still be used to find the answer. The only extra step required is flattening the generalized contact matrix into a 2D matrix of size $T \times T$ (as defined above, $T = K\prod_{p=1}^{m} V_p$). In these settings, the expression for the basic reproductive number becomes $R_0 = \frac{\Phi}{\Gamma}\rho(\tilde{\mathbf{G}})$ where $\tilde{\mathbf{G}} \in \mathbb{R}^{T\times T}$ is the generalized contact matrix whose elements are $\tilde{G}_{\mathbf{a},\mathbf{b}} = \frac{G_{\mathbf{a},\mathbf{b}}}{N_{\mathbf{b}}}N_{\mathbf{a}}$. $N_{\mathbf{a}}$ and $N_{\mathbf{b}}$ describe the number of individuals in subgroups $\mathbf{a}$ and $\mathbf{b}$ respectively. We can express this as the product of two matrices $\tilde{\mathbf{G}} = \mathbf{R_G}\mathbf{N_G^{-1}}$, where $\mathbf{N_G}$ is a diagonal matrix describing the number of individuals in each subgroup and $\mathbf{R_G} = \mathbf{N_G}\mathbf{G}$ is a symmetric matrix describing the total number of contacts between any pairs of subgroups.

The expressions for R_0 for single-attribute and generalized contact matrices are analogous. They both hinge on the spectral radius of a given matrix whose size and composition are generally different. Does this imply that the value of R_0 is also different? In other words, what happens to R_0 when we vary the number of variables used to stratify contact patterns? To answer this question, we can study what happens to R_0 when we increase the number of dimensions by 1. For example, let us consider the simplest case where we move from a single-attribute contact matrix to its generalization with an additional variable. In this case, but without loss of generality, the following theorem holds:

Theorem 1 (Spectral radius of generalized contact matrices). *Let* $\tilde{\mathbf{C}} = \mathbf{R}\mathbf{N}^{-1} \in \mathbb{R}^{K\times K}$ *be a single-attribute contact matrix where* $\mathbf{R}$ *is a symmetric, non-negative matrix, and* $\mathbf{N}$ *is a diagonal matrix, with positive diagonal elements. Let* $\tilde{\mathbf{G}} = \mathbf{R_G}\mathbf{N_G}^{-1} \in \mathbb{R}^{KV_1\times KV_1}$ *be a two-attribute generalization of* $\tilde{\mathbf{C}}$ *defined such that* $\sum_{\alpha,\beta=1}^{V_1}\left(R_G\right)_{i\alpha,j\beta} = R_{ij}$ *and* $\sum_{\alpha,\beta=1}^{V_1}\left(N_G\right)_{i\alpha,j\beta} = N_{ij}$ *with* $\mathbf{R_G}$ *being a symmetric, non-negative matrix and* $\mathbf{N_G}$ *being a diagonal matrix with positive diagonal elements. By denoting with* $\rho(\mathbf{M})$ *the spectral radius of a generic matrix* $\mathbf{M}$, *it follows* $\rho(\tilde{\mathbf{G}}) \geq \rho(\tilde{\mathbf{C}})$.

While the theorem explicitly treats a generalization obtained by shifting from one to two dimensions, the result holds for shifts to higher dimensions. In other words, adding any further attribute in the definition of the generalized contact matrix cannot decrease its spectral radius. The theorem does not make any assumption on the sizes K, V_1, and it can be easily adapted to generalized contact matrices with multiple dimensions. We discuss this extension in the Supplementary Materials, alongside the theorem's proof.

The theorem implies that a generalized contact matrix stratified across $m + 1$ dimensions has a spectral radius (hence basic reproductive number R_0) equal or higher than its s-dimensional counterpart ($1 \leq s \leq m$). As we increase the number of dimensions used to describe contact patterns, R_0 cannot decrease. Hence, the theorem defines a lower bound for R_0. As a corollary, it can be proven that the strict equality can be attained under the random mixing hypothesis, i.e., when the contacts across the additional dimensions are set proportional to the product of the subpopulation sizes (see the Supplementary Materials). Consequently, generalizing contact matrices by knowing only the number of individuals in a given subgroup via a random mixing assumption does not change the epidemic dynamics but increases the complexity of the model. In general however, as shown in the next sections, the variables used to capture the stratification of contacts and the way interactions are aggregated (i.e., by age or other dimensions) might affect the description of epidemic processes, especially in the presence of nontrivial correlations between explicit and implicit (i.e., aggregated) variables. Hence, the estimation of the spreading potential of a disease via a model is a function not only of the total number of contacts but also of how these contacts are arranged across groups. This observation is one of the key insights from network-based epidemiology: The epidemic threshold (i.e., R_0) in two populations with the same number of individuals and number of contacts is drastically influenced by the way interactions are organized (i.e., the topology of the network) (*39*).

Numerical simulations: Synthetic data

First, we test the analytical results obtained in the previous section by considering synthetic generalized contact matrices. To ground the results with empirical observations, we build on prototypical contact matrices, where interactions are stratified only according to age. Here, we rely on empirical data from Hungary using a pre–COVID-19 contact matrix stratified by age (*40*). We report analogous findings for a different country in the Supplementary Materials. We create generalized contact matrices by adding more dimensions

to the empirical matrices and defining the contact rates in the added groups with a simple model. We explore two cases. In the first case, contact rates for the additional dimensions are set to be proportional to the product of the population sizes of the different groups. This is the aforementioned random mixing assumption. In the second case, instead, we investigate scenarios where parameters define contact rates among subgroups. This allows us to introduce correlations in contact patterns, such as assortativity, where members in a given group (e.g., in an SES class) are more likely to contact people in the same group. Furthermore, in the second case, we introduce activity parameters to adjust the activity (i.e., the share of contacts) of population groups. As shown below, when considering empirical data from Hungary with an additional dimension, nontrivial correlations and heterogeneities appear. We refer the reader to the Materials and Methods and the Supplementary Materials for details about constructing the synthetic generalized contact matrices. In Fig. 2A, we display the empirical contact matrix **C** for Hungary, which stratifies interactions in $K = 8$ age brackets. While until the 45- to 60-year age group, the highest values of contact rates are within the same age bracket (i.e., diagonal elements), we observe strong off-diagonal values for age groups that range between 15 and 60 years. In Fig. 2B, we show the flattened 2D representation of a generalized contact matrix where we have a second dimension, besides age. We imagine a simple case where the second dimension contains three groups, i.e., $V_1 = 3$. For simplicity, we assume that 35, 45, and 20% of the population belong to these three categories across all age groups. The matrix is formed by $K \times V_1 = 24$ distinct groups.

Random mixing scenario

First, we assume contacts—when it comes to the second dimension—to be proportional to the product of the population size in each group. Hence, they are the expected values of a random mixing process. As mentioned above, this assumption leads to a generalized contact matrix with the same spectral radius as the original matrix. The effects of this property on an epidemic model, used to study the spread of a virus in the population, are shown in Fig. 2C. We plot the attack rate (i.e., epidemic size) as a function of R_0 for (i) a model fed only with the contact matrix C_{ij} (gray crosses), (ii) a model fed with the generalized contact matrix $G_{\mathbf{a},\mathbf{b}}$ described above (green squares), and (iii) a model fed with the matrix $C_{\alpha\beta}$ where contacts are stratified only according to the second dimension (red circles). As a result, we observe, in perfect agreement with the analytical formulation, that $R_0 = 1$ divides the phase space into two regions. For values below this threshold, the disease does not spread in any of the three models. Only for values larger than one, the disease infects a finite fraction of the population. We observe that the threshold and the attack rates for different values of R_0 are equal in the first two models. As expected, when assuming random mixing along the second contact dimension, the results of a model that either neglects or considers such additional dimension are equal. Hence, the fraction of infected individuals in the groups of the additional category follows the same trajectory (see Fig. 2D). We note, however, clear differences concerning the third model (red circles) that features a 3 × 3 contact matrix capturing the stratification of contacts only for the additional category. A description of the epidemic in these settings leads to higher attack rates for any given $R_0 > 1$. This observation confirms

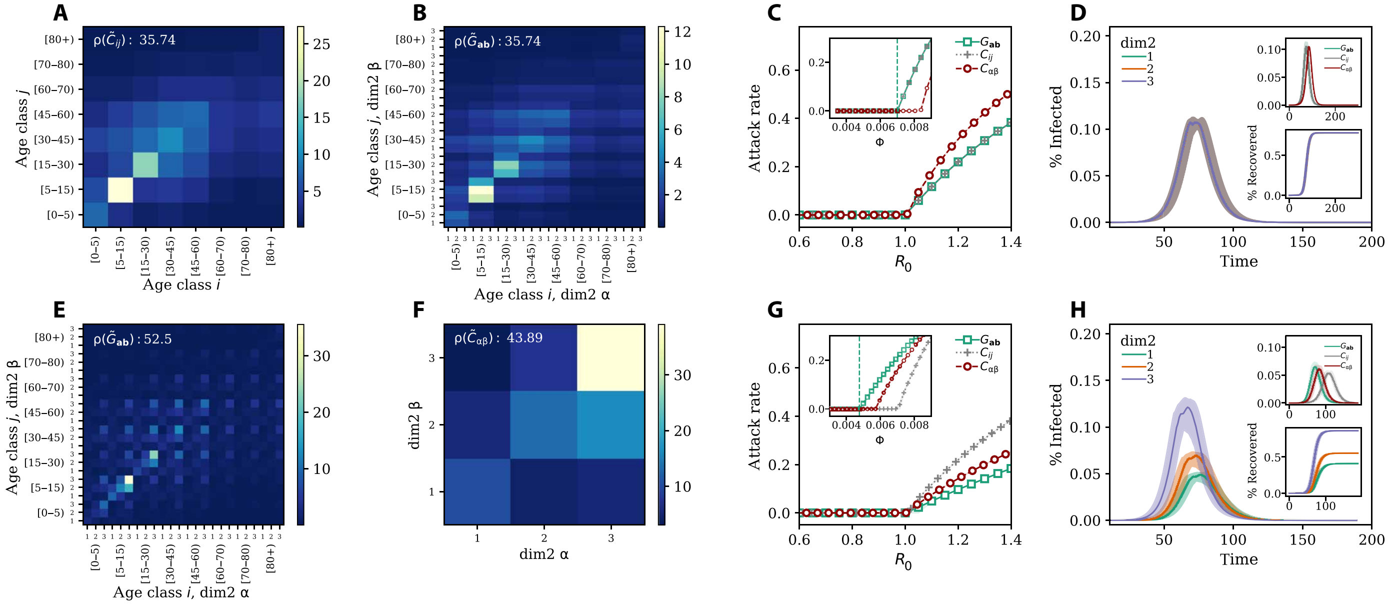

Fig. 2. Impact of generalized contact matrices on epidemic spreading. (**A**) Example of a classic age contact matrix **C** (8 × 8); (**B**) and (**E**) generalized contact matrices with an additional dimension with three groups **G** (24 × 24) in the case of random mixing (B) and (E) assortative mixing in the second dimension; (**F**) depicts the case when we integrate the generalized contact matrix over all age classes. The ρ values indicate the spectral radius corresponding to each matrix. In (**C**), we show the attack rate as a function of R_0 (main figure) and disease transmissibility (inset). In (**D**), we show, in the case of random mixing, the prevalence (main figure and top inset) and the fraction of recovered (bottom inset) as function of time. The colored lines and shaded areas in the main panel and in the bottom inset represent, respectively, the prevalence and fraction of recovered individuals in the three groups of the additional category (i.e., dim2); (**G**) and (**H**) are as the previous two plots but in case of assortative mixing with different levels of activity. Results refer to the median of 500 runs. Epidemiological parameters: $\Gamma = 0.25$, $\Psi = 0.4$, and $R_0 = 2.7$. Simulations start with a number $I_0 = 100$ of initial infectious seeds.

how within the same population the chosen stratification of contacts might affect the description of an epidemic unfolding in the system.

In the inset of Fig. 2C, we show the attack rate as a function of the transmissibility Φ. The vertical dashed line denotes the analytical critical value of Φ obtained by setting the expression for R_0 equal to one for the model featuring the generalized contact matrix. The plot shows the equivalence between the first model and the second as well as the difference with respect to the third. More precisely, the critical value of Φ in the model featuring the matrix $C_{\alpha\beta}$ is larger than the other two models. This implies a smaller value of R_0, for a given value of Φ. The result is in line with the lower-bound theorem (Theorem 1). $C_{\alpha,\beta}$ is a lower dimensional representation of $\mathbf{G}$. Hence, its spectral radius is either equal or smaller. It is important to stress, however, that $C_{\alpha\beta}$ is obtained from $\mathbf{G}$ by aggregating across all age-dependent correlations. Hence, the corollary of the lower bound theorem, which is valid when the stratification along the additional dimension is obtained by random mixing, does not hold in general.

Assortative scenario

We now consider the second scenario: assortative mixing. In Fig. 2E, we display the 2D flattened generalized contact matrix obtained by assuming that 60, 50, and 65% of the contacts in the first, second, and third groups of the additional category take place within each group, this way inducing assortativity. Furthermore, we assume some levels of heterogeneity also in the activity of the different groups setting as 20, 40, and 40% the share of contacts of the three groups, respectively. Even a qualitative visual inspection of the generalized contact matrix shows how the introduction of assortativity and activity changes the contact rates (see Fig. 2E) with respect to the previous case of random mixing (Fig. 2B). This is confirmed by the value of the spectral radius which is about 46% larger, increasing from $\rho(\tilde{G}_{ab}) = 35.74$ to 52.5. In Fig. 2F, we show the matrix $C_{\alpha\beta}$ obtained after integrating the generalized contact matrix over all age classes. As imposed by construction, the third group is characterized by a higher assortativity than the others. This group represents the smallest fraction of the population and is the most active (together with the second group). These characteristics explain the high diagonal value in the matrix describing the contact rate between individuals in that group. As a result, the spectral radius is 24% higher $[\rho(\tilde{C}_{\alpha\beta}) = 43.89]$ than the one of the contact matrix stratified by age [with $\rho(\tilde{C}_{ij}) = 35.74$]. The impact of the different contact matrices on the simulated attack rate is shown in Fig. 2G where we plot it as a function of R_0. The figure shows in each case that the critical point falls at $R_0 = 1$, thus the analytical solutions match the numerical simulations very well.

Contrary to the previous case of random mixing, the attack rates of the model that is informed with the contact matrix C_{ij} and the one with the generalized matrix $G_{\mathbf{a},\mathbf{b}}$ are different for $R_0 > 1$. For a given R_0 the strong assortativity of contacts constrains the spreading of the disease, resulting in a smaller infected population fraction (see the green squares). Furthermore, the most active group is also the smallest one in this setting. A similar behavior is observed in contagion processes unfolding on explicit contact networks organized in clusters, where the local topological correlations might slow down the spreading of contagion processes that in turn do not evolve into an endemic state (i.e., SIR-like dynamics) (*41*). If the contacts are stratified according to the second dimension only (red circles), the attack rates are closer to those emerging from the generalized contact matrices (green squares). In the inset of the figure, we show the attack rate as a function of the transmissibility Φ. The vertical dashed line denotes the theoretical prediction of its critical value when considering the generalized contact matrices. Because of the differences in the spectral radius of the various matrices, the critical values of Φ do not coincide. In the setting considered here, neglecting the assortativity and activity across the second dimension in favor of simpler representations focused only on age or the additional variable leads to a possibly large underestimation of the critical value of Φ. Assortativity and activity also introduce variations in the disease burden across the secondary dimension. Figure 2H shows how, in these settings, individuals in the third class are affected earlier and more intensely than the others. This is due to their high activity and assortativity. The result is confirmed by looking at the evolution of the fraction of recovered as a function of time (bottom inset of Fig. 2H) where we see how individuals in the third class are substantially more affected by the disease. Last, in the top inset of Fig. 2H, we show the prevalence for three models for a fixed value of $R_0 = 2.7$. Contact matrices stratified only by age feature a smaller and later peak with respect to the other two. Even though these results are driven by the assumptions made when building the generalized contact matrix, they show how modeling outcomes, based on models that include or neglect further stratification of contacts besides age, might be extremely different. These differences cannot be observed a posteriori. For example, estimating the prevalence in a subgroup, by simply considering its share of the population, cannot account for differences in peak timing or heterogenities in epidemic outcomes. Moreover, the corollary of the lower-bound theorem highlights how even more refined attempts to include extra dimensions into epidemic models might not be helpful. In the absence of empirical contact data, it might be tempting to use subgroup sizes and build generalized contact matrices via a random mixing assumption. However, our results formally demonstrate that this would inevitably lead to the same epidemic dynamics of lower dimensional representations. In the Supplementary Materials, we report an extensive exploration of the parameter space, confirming our analytical solutions' validity. We also include scenarios that consider additional dimensions. The results confirm that the analytical expression for R_0 provides a good description of the epidemic dynamics even in generalized contact matrices with three dimensions.

Modeling the adoption of NPIs

As mentioned above, epidemic models featuring generalized contact matrices are more expressive than traditional approaches. They allow capturing possible heterogeneities in behaviors that might span from NPI adoption to vaccination uptake across diverse population subgroups. The COVID-19 pandemic has given a stark reminder that the ability to comply with NPIs or vaccine uptake is far from homogeneous. They correlate and are influenced by many socioeconomic variables (*17*, *18*, *24*, *34*). Standard models can only partially describe such heterogeneities, considering, for instance, contact reductions as a function of age and location (*42*).

To showcase the potential of generalized contact matrices in this context, we explore hypothetical scenarios where a given population, featuring an additional dimension beside age, changes behavior following the introduction of NPIs. We assume the ability to reduce contacts to be associated with the membership to a particular population group. We start with a pre-epidemic baseline where 33, 50, and 60% of the contacts in the first, second, and third groups of the additional category take place within each group. In

the baseline 25, 45, and 30% are the shares of contacts of the three groups. For simplicity, we assume an equal population distribution across the three groups. In Fig. 3A, we show the prevalence of a disease that spreads unmitigated by any NPI in our baseline population. Individuals in the second group are the most affected because of their higher activity. This is confirmed in the bottom inset where we plot the fraction of recovered as a function of time. The second group is more affected than the other two. The top inset shows the estimation of the overall prevalence considering, as before, the three epidemic models featuring generalized contact matrices (green line), age-stratified matrices (gray line), and contact matrices stratified only for the second dimension (red line). In these settings, the first model peaks a bit earlier, but the three curves are overall very similar.

Then, we imagine two scenarios where at time $t^* = 50$, because of NPIs, the population changes behaviors. We refer to the Supplementary Materials for the details about the implementation. In Fig. 3B, we show what would happen to the epidemic in case of a homogeneous reduction scenario when all groups would be able to reduce their contacts by 35%. The impact of the NPIs is quite strong and induces a clear reduction of the peak across the three groups of the population. The top inset of Fig. 3B shows how in this scenario the overall prevalence, estimated with a model featuring generalized contact matrices (green line), peaks earlier and higher than with the other two. The model fed with age-stratified contact matrices (gray line) is very similar. The third model (red line) instead is rather different as the peak is not only lower but also delayed. This is another reminder of how the representation of contacts, the choice of which variables are considered or integrated, might affect the estimation of the epidemic dynamics. Moreover, as noted above, such differences in peak time cannot be observed by considering the additional dimensions only at posteriori. In the bottom panel of Fig. 3B, we plot the fraction of recovered population as function of time. We observe how the implementation of NPIs reduce the burden of the disease with respect to the baseline, but as before individuals in the second subgroup of the additional dimension are the most affected.

In Fig. 3C, we explore a scenario where the first group (e.g., representing for example the lowest SES class) is not able to protect itself as the other two manage to afford. We model this by reducing the overall number of contacts by 35% but assuming that the NPIs introduce changes in both assortativity and activity. We imagine that, because of the NPIs, 50, 60, and 70% of the contacts in the first, second, and third groups of the additional category take place within each group. Hence, across the groups, assortativity increases but more substantially for the second and third group. Furthermore, we imagine that the NPIs shift activities to 37, 37, and 26% across the three groups. Hence, while activity decreases for the second and third groups, it relatively increases for the first. Observing the prevalence curves, we see how the NPIs bring a general reduction, but the changes in the contact patterns affect the first group more negatively. We observe a switch from a baseline where this group was the least affected to a scenario where it becomes the most affected (together with the third group). This is confirmed in the bottom inset of the figure where we plot the fraction of recovered individuals as a function of time for the three subgroups in the additional dimension. The top inset of the figure shows the overall prevalence for the three models that is very close to the previous.

Numerical simulations: Empirical data

We applied the model to empirical data describing social contacts stratified by age and one SES variable (i.e., self-perceived wealth with respect to the average) in Hungary. The data have been collected via computer-assisted surveys from 1000 respondents describing a representative sample of the Hungarian adult population in terms of gender, age, education level, and type of settlement (*40*). We refer the reader to Materials and Methods and the Supplementary Materials for more details about the data and its collection. In Fig. 4A, we show the traditional contact matrix that focuses only on age. Though similar, the structure of contact patterns differs with respect to the equivalent matrix for the same country shown above in Fig. 2A. This discrepancy is due to the different periods these matrices represent. While the matrix in Fig. 2A has been collected before the COVID-19 pandemic, data in Fig. 4A record typical contact patterns during the pandemic, specifically in June 2022. Although at that time the vaccination campaign was in full swing and the number of confirmed cases and deaths was relatively low, the previous wave had peaked only a few months before and some level of social distancing was still in place (for more details about the construction and normalization of this matrix, see the Supplementary Materials). The matrix confirms that children and young adults are the most

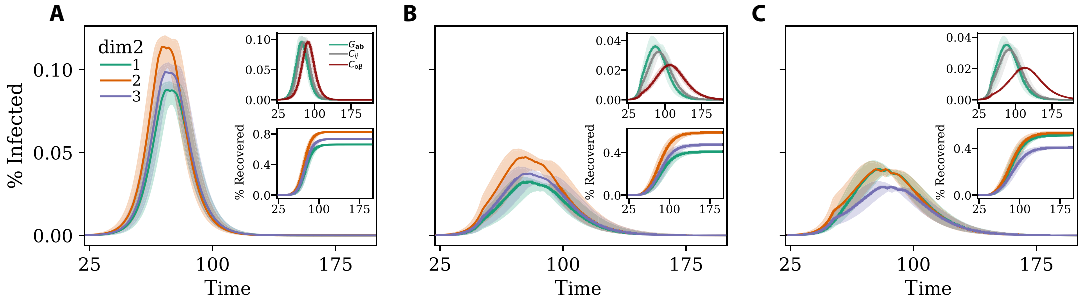

Fig. 3. Modeling the adoption of NPIs. Disease prevalence in (**A**) baseline scenario (**B**) and (**C**) with NPIs introduced at $t^* = 50$ that induce a reduction of 35% in total number of contacts with respect to the baseline scenario. In the case of (B), the three groups reduce the number of contacts equally, while in (C), the NPIs induce a redistribution of the number of contacts. Curves in the main plot indicate the prevalence for the three groups of the additional dimension. The top inset shows the prevalence for models featuring generalized contact matrices (green line), standard age-stratified contact matrices (gray line), and contact matrices stratified by the additional dimension (red line). The bottom inset shows the fraction of recovered as a function of time for the three groups of the additional dimension. Results refer to the median of 500 runs with confidence intervals. Epidemiological parameters: $\Gamma = 0.25$, $\Psi = 0.4$, and $R_0 = 2.7$. Simulations start with $I_0 = 100$ initial infectious seeds.

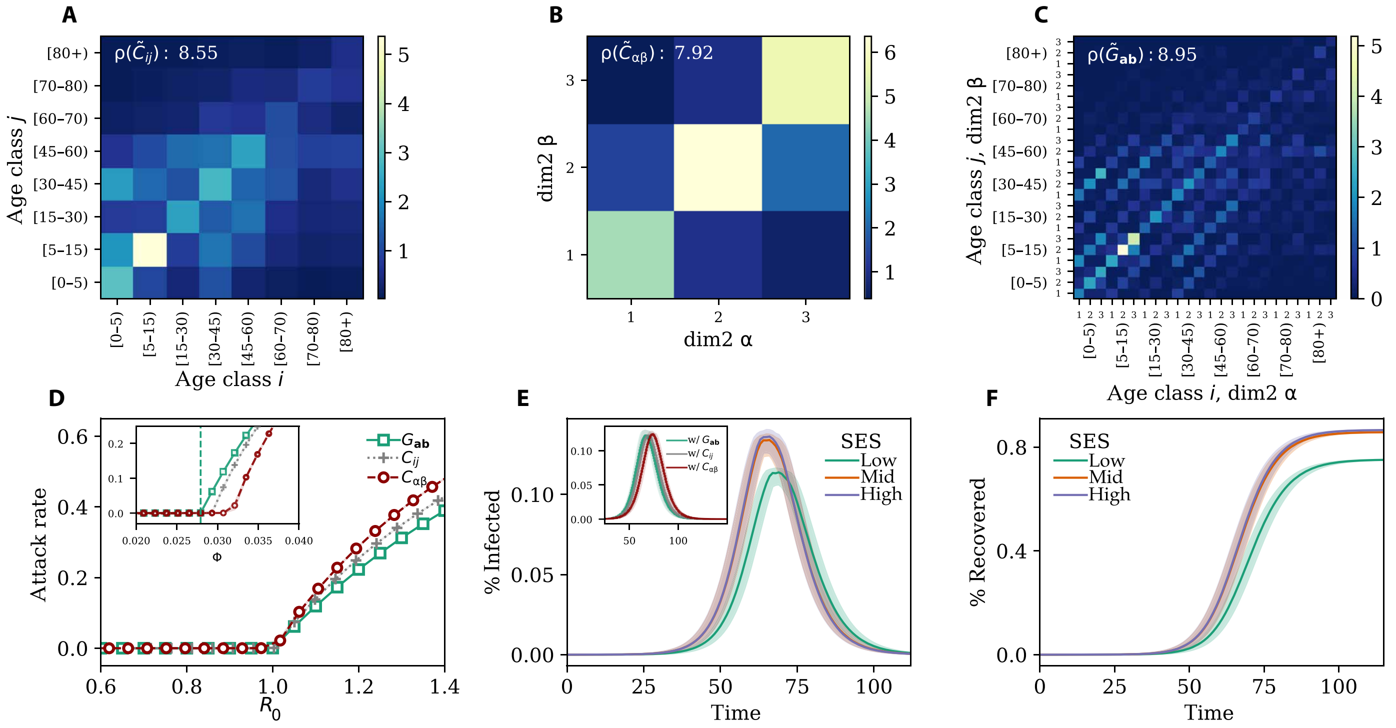

Fig. 4. Empirical generalized contact matrices. (**A**) Age contact matrix **C** (8 × 8); (**B**) SES contact matrix $C_{\alpha\beta}$ (3 × 3) and (**C**) generalized contact matrix with age and SES **G** (24 × 24); (**D**) attack rate as a function of R_0 (main figure) and transmissibility (inset); (**E**) disease prevalence in different SES groups (main figure) and different representation of the contact patterns (inset); (**F**) fraction of recovered individuals in different SES groups. In (A) to (C), ρ indicates the corresponding spectral radius. Results in (D) and (E) refer to the median of 500 runs. Epidemiological parameters: Γ = 0.25, Ψ = 0.4, and R_0 = 2.7. Simulations start with I_0 = 100 initial infectious seeds. The matrices have been computed using the contact diaries coming from the MASZK data collected in Hungary during June 2022.

active and that their interactions are largely assortative. However, the matrix features a rather smaller spectral radius with respect to the prepandemic contact matrix. This highlights the reduction in contacts caused by the COVID-19 emergency. In Fig. 4B, we show the stratification of contacts considering only the SES variable, which divides the population into three SES groups with Low, Medium, and High wealth. Similar to other studies (*43*, *44*), we observe a strong diagonal component, confirming high levels of assortativity within SES groups. The population experiencing mid-low SES features lower assortativity and activity. Furthermore, the off-diagonal elements display higher levels of segregation for people experiencing low SES, as individuals experiencing mid and high SES tend to interact more with each other. In Fig. 4C, we show the flattened representation of the generalized contact matrix. $\rho(\tilde{G}_{\mathbf{a,b}})$ is higher than the other two, but the difference is clearly more limited with respect to the synthetic case we discussed in the previous section. In Fig. 4D, we show the attack rate as a function of R_0 for a disease spreading in a susceptible population described with each of the three contact matrices discussed. It is important to stress that these simulations do not aim to reproduce the evolution of the COVID-19 pandemic in Hungary. Instead, they describe a hypothetical disease spreading on different representations of the contact patterns measured in June 2022. Our goal is to showcase the possible variations in the description of an epidemic. The theoretical critical value for the basic reproductive number (being at $R_0 = 1$) well matches the numerical simulations. Furthermore, for a given value of $R_0 > 1$ the attack rate estimated with a model featuring the generalized contact matrix (green squares) is always smaller than any of the two other models. Also in this case, the more realistic description of contacts across multiple dimensions constrains the unfolding of the disease with respect to scenarios that neglect one of the two dimensions. However, the model featuring the contact matrix stratified by SES only (red circles) leads to higher attack rates than the model featuring an age-stratified contact matrix. The inset confirms the validity of the mathematical formulation and highlights how a higher spectral radius results in a lower critical value for Φ. In Fig. 4E, we plot the prevalence of the disease for the model with a generalized contact matrix, splitting the three SES groups. We observe that individuals experiencing high SES are the most affected in terms of contracted infections. This is due to their higher contact activity. Those experiencing mid-SES follow very closely, partially because of the strong interaction activities with the first group. Individuals experiencing low SES, instead, are affected later and are subject to a lower prevalence. In this scenario, where the population is not subject to further NPIs nor spontaneously changing behavior during the epidemic, the higher level of segregation experienced by the low-SES group has the silver lining effect of shielding that group from the epidemic, in accordance with empirical observations in the country (*45*). These results are confirmed in Fig. 4F where we plot the fraction of recovered as function of time for individuals in different SES groups. In the inset of Fig. 4E, we show the overall evolution of the disease obtained by modeling the epidemic with each of the three contact matrices. The plot confirms how the chosen representation of contacts influences the description of the disease. Considering different

numbers or types of dimensions, in this case, induces differences in the estimation of peak time, which is a key variable used to inform public health measures.

In the Supplementary Materials, we repeat the same analyses considering data collected at different time points of the COVID-19 pandemic, in April and November 2021. While the overall results confirm the picture that emerged here, models fed with standard and generalized contact matrices lead to estimations of the attack rates, for a given R_0, which are closer to each other. Nevertheless, the model featuring generalized contact matrices allows capturing heterogeneities in the prevalence across SES groups, which are invisible to standard approaches and to analyses conducted integrating SES dimensions only at posteriori.

DISCUSSION

In this study, we presented a mathematical epidemic modeling framework featuring generalized contact matrices, that is, contact matrices that are stratified according to multiple dimensions such as variables linked to SES. The goal of our work is to highlight the importance of moving beyond the traditional representation of mixing patterns, based on age-specific and/or context-specific contact rates, allowing for the development of structured epidemic models that incorporate multiple groups of the population at once.

First, we focused on the mathematical characterization of the modeling framework. We found that the basic reproductive number, R_0, of a model featuring generalized contact matrices can still be obtained analytically via the next-generation matrix approach developed for traditional compartmental models (*36*, *37*). We then introduced a lower-bound theorem proving how the generalization to multiple dimensions leads to a value of R_0, which is necessarily equal to or larger than for models featuring fewer grouping dimensions. We also found a corollary of the theorem proving that the R_0 of a model fed with generalized contact matrices, obtained via a random mixing approximation for the additional dimensions, is necessarily the same as the correspondent value for models fed with lower dimensional contact matrices. We showed, numerically, how nontrivial correlations of contacts across and within groups, such as assortativity and heterogeneous activity, increase the value of R_0. We then explored how the modeling framework can be used to integrate and consider explicitly SES dimensions. Through numerical simulations, we showed how models featuring generalized contact matrices better capture heterogeneous behaviors across population subgroups, such as varying rates of adherence to NPIs across socioeconomic strata, as observed in reality (*27*, *29*, *30*, *46*). By using both synthetic and empirical data from Hungary, we demonstrated how neglecting SES dimensions in favor of simpler representations of social contacts may result in large misrepresentations of attack rates, epidemic thresholds, and disease burden across different population subgroups. Moreover, our results highlight how these misrepresentations cannot be simply corrected by integrating SES dimensions a posteriori.

The COVID-19 pandemic has tragically reminded us that health emergencies do not affect populations equally and that social, economic, and cultural forces fundamentally shape the outcome of disease outbreaks, reflecting the socioeconomic inequalities of the society at large (*47*–*50*). These observations are in stark contrast with the traditional structure of epidemic models, which often neglect all variables but age as the key demographic feature defining disease transmission in a population (*38*). Recent studies have highlighted the urgent need for modeling approaches that can account for the multiple social dimensions that define the risk of infection and infection outcomes (*17*, *18*, *20*). In the wake of the COVID-19 pandemic, research studies have introduced social contact matrices stratified by alternative demographic groups, such as racial and ethnic subpopulations (*51*), but usually without considering more than one dimension at once. As an alternative, scholars have developed large-scale individual-based models that reconstruct synthetic populations of millions of agents with extreme realism, including many key socioeconomic characteristics (*16*, *52*–*54*). These models, however, require the availability of highly resolved mobility data derived, e.g., from mobile phone logs, and large computational infrastructures for data processing and simulations. Previous studies have compared different data representation methods, ranging from fully homogeneous mixing to temporal networks, to identify the relevant trade-offs between compactness and realism (*55*–*58*). Our work attempts to strike a balance between traditional approaches that are too simplistic and the complexity of large-scale synthetic populations, providing a general and flexible scheme to define epidemic models with multiple interacting population subgroups.

As for any structured epidemic modeling approach, our model requires data to parameterize social contact rates across subgroups. Social contact surveys have been and will continue to be an important asset for the study of mixing behaviors both in normal conditions and during epidemic outbreaks (*2*, *35*, *59*–*63*). Future contact surveys could include additional demographic and socioeconomic dimensions beyond the usual age/gender components. The work we presented here shows how these dimensions can be easily integrated by generalizing traditional epidemic models. In some cases, large-scale contact surveys including several population attributes can be unfeasible, especially in resource-poor settings. Alternative approaches could infer contact patterns from the analysis of demographic data that are routinely collected by census surveys (*5*, *12*). Other proxies of contact rates, derived from mobility data, could be similarly used to infer contact patterns among different socioeconomic groups, as demonstrated by recent studies focusing on experienced segregation in large US metropolitan areas (*64*, *65*).

It is important to mention the limitations of our work and the directions for future developments of our study. Admittedly, the model we developed to generate synthetic generalized contact matrices does not attempt to reproduce empirical observations from real data but rather to provide a general test bed for investigation. This choice was guided by the lack of available data about the stratification of contacts across multiple dimensions in different countries. As described, we had access to empirical data only for Hungary. Arguably, one country is not enough to develop a general model, and more work is needed to design and test other approaches. Similarly, the methodology we used to simulate the effects of NPIs was driven by simplicity rather than realism. We intended to showcase the potential of our model to capture possible heterogeneities in behaviors rather than reality. Data from Hungary contained limited information about the social contacts of children. Hence, we had to introduce a few assumptions about their structure. Another important assumption we made is about the independence of age and other socioeconomic dimensions. While this assumption was necessary to showcase our methodology in simple terms, future work is needed to investigate the impact of such correlations on the description of epidemic processes and to explore the applicability/extension of methods for dimensionality reduction.

Last, it is important to acknowledge important ethical and privacy concerns linked with data collection efforts that inspect individuals about several variables. By increasing the number of dimensions the size of each surveyed group rapidly decreases. Risks of reidentification are real and of particular concern, especially for minorities and underrepresented groups (*66*). Progresses in the direction we have proposed here are necessarily linked to progresses in data collection and dissemination that reconcile the development of better public health tools on the one side with privacy rights on the other (*67*). Arguably, it is this unresolved tension that, among other reasons, has so far limited the collection and sharing of contact survey data including more dimensions besides age. Nevertheless, the data we use demonstrate that such data collection is possible in an anonymous and privacy-protected way.

Overall, our work contributes to the literature by attempting to bring socioeconomic and other dimensions to the forefront of epidemic modeling. Tackling this issue is crucial for developing more precise descriptions of epidemics and thus to design better strategies to contain them.

MATERIALS AND METHODS

Synthetic generalized contact matrices

We developed a model to build synthetic generalized contact matrices $G_{\mathbf{a},\mathbf{b}}$. For simplicity and clarity of exposition, let us consider only two dimensions: age and an additional variable, for example one SES indicator. Let us denote with K the number of age brackets and V_1 the number of subgroups in the second dimension. In these settings, $\mathbf{a} = (i, \alpha)$ and $\mathbf{b} = (j, \beta)$. The indices i and j refer to the age group while α and β to the SES of the ego and the alter, respectively. The model takes as input a real contact matrix $\mathbf{C}$ describing the contact rates between age brackets. As mentioned above, we can write $\mathbf{C} = \mathbf{N}^{-1}\mathbf{R}$ where $\mathbf{N}$ is a diagonal matrix (with positive diagonal elements) describing the number of individuals in each age bracket, and $\mathbf{R}$ is a symmetric matrix describing the total number of contacts between age groups in a given period. The generalized contact matrices $\mathbf{G}$ can be expressed as $\mathbf{G} = \mathbf{N}_{\mathbf{G}}^{-1}\mathbf{R}_{\mathbf{G}}$ where analogously $\mathbf{N}_{\mathbf{G}}$ is a diagonal matrix (with positive diagonal elements) describing the number of individual in each $\mathbf{a}$ and $\mathbf{R}_{\mathbf{G}}$ is a symmetric matrix capturing the number of total contacts between any two $\mathbf{a}$ and $\mathbf{b}$ in a given period. To build generalized contact matrices, we need to define these two matrices. The first can be obtained by assuming a given population distribution for each $\mathbf{a}$. The second requires more attention. The construction needs to respect two properties. The first is symmetry: $(R_G)_{i\alpha,j\beta} = (R_G)_{j\beta,i\alpha}$. In words, the number of contacts that individuals in age group i and SES α have with individuals in age group j and SES β must be equal to the number of contacts that individuals in age group j and SES β have with individuals in age group i and SES α. This property implies that, for all $i = j$, $(R_G)_{i\alpha,i\beta} = (R_G)_{i\beta,i\alpha}$. In general, this is not the case for $i \neq j$. The second property is that $R_{ij} = \sum_{\alpha,\beta}^{V_1} \left(R_G\right)_{i\alpha,j\beta}$. In words, by integrating across the second dimension, we must obtained the initial matrix $\mathbf{R}$. For a given pair i and j, $(R_G)_{i\alpha,j\beta}$ is of size $V_1 \times V_1$. Hence, for any $i \neq j$, we need a model to set a number $W = V_1^2 - 1$ of elements in the matrix. Note how the minus one stems from the constraint introduced by the second property. For all $i = j$ instead, the symmetry of the matrix is such that we need to set only $Y = V_1 + \frac{V_1(V_1-1)}{2} - 1$ elements. The first V_1 comes from the diagonal (i.e., $\alpha = \beta$), the $\frac{V_1(V_1-1)}{2}$ are instead the off-diagonal values, and the minus one as before is due to the second property of such matrices. To reduce the number of parameters, we assume an independence between age and other dimensions. In this case, because $W > Y$ for all $V_1 \geq 2$, we can define the generalized contact matrix by setting W values. These can be obtained via a simple model where, for any i and j pair, (i) each SES α is responsible for a fraction P_α of R_{ij} connections, and (ii) a fraction q_α of these are on the diagonal (i.e., in-group connections) and $1 - q_\alpha$ are instead off diagonal. This parameter controls the assortativity of connections within each group. The fractions P_α and q_α are input parameters. By setting $V_1 - 1$ values P_α (the minus one stems from the constraint $\sum_\alpha^{V_1} P_\alpha = 1$) and V_1 values q_α, we obtain a model with $2V_1 - 1$ parameters. In case this number is equal to W, the constraints imposed by our assumptions allow us to define all the entries of the generalized contact matrix. If instead $W > 2V_1 - 1$ (guaranteed for any $V_1 \geq 3$), other $W - 2V_1 + 1$ free parameters (besides the values of P_α and q_α) are required for each i and j pair. We refer the reader to the Supplementary Materials for more details about synthetic generalized contact matrices.

Empirical generalized contact matrices

We built generalized contact matrices stratified in two dimensions by using real data from the MASZK study (*40*, *68*) (for more information about the dataset, see the Supplementary Materials). The data provided us with a range of information about the anonymous participants including their perceived wealth with respect to the average, which is one SES variable we rely on. Information on social interactions have been collected in two different ways: (i) in an aggregate form, such that each participant reported the number of contacts they had with individuals in each of the eight age brackets considered and (ii) in a diary in which each participant listed one by one the interactions they had on a given day by reporting some meta information of the contactee such as their age and SES. In particular, the average number of contacts of an individual in age class i, and SES α with an individual in age class j and SES β is $G_{\mathbf{a},\mathbf{b}}$ where $\mathbf{a} = (i, \alpha)$ and $\mathbf{b} = (j, \beta)$. However, the MASZK data provided us with diary information only for the adult population (individuals older than 15 years old). For the children, their average number of contacts is available only in the aggregate form for the eight age brackets considered. Thus, we assigned the average number of contactees to the different SES as follow: $G_{i\alpha,j\beta} = G_{i\alpha,j}u_{\alpha\beta}$, where $G_{i\alpha,j}$ is the average number of contacts that individual of age group i and SES α has with all the individuals of age group j, and $u_{\alpha\beta}$ is a parameter that controls how these contacts are distributed among individuals of different SES.

Numerical simulations

To investigate the effect of the generalized contact matrices $G_{\mathbf{a},\mathbf{b}}$ on the transmission dynamics, we developed a stochastic, discrete-time, compartmental model where the transitions among compartments are simulated through chain binomial processes. In particular, at time step t, the number of individuals in group $\mathbf{a}$ and compartment X transiting to compartment Y is sampled from $PrBin[X_{\mathbf{a}}(t), p_{X\mathbf{a}\rightarrow Y\mathbf{a}}(t)]$, where $p_{X\mathbf{a}\rightarrow Y\mathbf{a}}(t)$ is the transition probability.

Supplementary Materials

This PDF file includes:

Supplementary Text

Figs. S1 to S6
Tables S1 and S2
References

REFERENCES AND NOTES

1. W. J. Edmunds, C. J. O'callaghan, D. J. Nokes, Who mixes with whom? A method to determine the contact patterns of adults that may lead to the spread of airborne infections. *Proc. Biol. Sci.* **264**, 949–957 (1997).
2. J. Mossong, N. Hens, M. Jit, P. Beutels, K. Auranen, R. Mikolajczyk, M. Massari, S. Salmaso, G. S. Tomba, J. Wallinga, J. Heijne, M. Sadkowska-Todys, M. Rosinska, W. J. Edmunds, Social contacts and mixing patterns relevant to the spread of infectious diseases. *PLOS Med.* **5**, e74 (2008).
3. K. Prem, A. R. Cook, M. Jit, Projecting social contact matrices in 152 countries using contact surveys and demographic data. *PLOS Comput. Biol.* **13**, e1005697 (2017).
4. P. Rohani, X. Zhong, A. A. King, Contact network structure explains the changing epidemiology of pertussis. *Science* **330**, 982–985 (2010).
5. D. Mistry, M. Litvinova, A. Pastore, Y. Piontti, M. Chinazzi, L. Fumanelli, M. F. C. Gomes, S. A. Haque, Q.-H. Liu, K. Mu, X. Xiong, M. E. Halloran, I. M. Longini, S. Merler, M. Ajelli, A. Vespignani, Inferring high-resolution human mixing patterns for disease modeling. *Nat. Commun.* **12**, 323 (2021).
6. J. Wallinga, P. Teunis, M. Kretzschmar, Using data on social contacts to estimate age-specific transmission parameters for respiratory-spread infectious agents. *Am. J. Epidemiol.* **164**, 936–944 (2006).
7. T. Hoang, P. Coletti, A. Melegaro, J. Wallinga, C. G. Grijalva, J. W. Edmunds, P. Beutels, N. Hens, A systematic review of social contact surveys to inform transmission models of close-contact infections. *Epidemiology* **30**, 723 (2019).
8. L. D. Amico, J. Kleynhans, L. Gauvin, M. Tizzoni, L. Ozella, M. Makhasi, N. Wolter, B. Language, R. G. Wagner, C. Cohen, S. Tempia, C. Cattuto, Estimating household contact matrices structure from easily collectable metadata. *PLOS ONE* **19**, 1–13 (2024).
9. R. M. Anderson, R. M. May, Age-related changes in the rate of disease transmission: Implications for the design of vaccination programmes. *Epidemiol. Infect.* **94**, 365–436 (1985).
10. R. M. Anderson, R. M. May, Vaccination against rubella and measles: Quantitative investigations of different policies. *Epidemiol. Infect.* **90**, 259–325 (1983).
11. R. Verity, L. C. Okell, I. Dorigatti, P. Winskill, C. Whittaker, N. Imai, G. Cuomo-Dannenburg, H. Thompson, P. G. T. Walker, F. Han, A. Dighe, J. T. Griffin, M. Baguelin, S. Bhatia, A. Boonyasiri, A. Cori, Z. Cucunubá, R. FitzJohn, K. Gaythorpe, W. Green, A. Hamlet, W. Hinsley, D. Laydon, G. Nedjati-Gilani, S. Riley, S. van Elsland, E. Volz, H. Wang, Y. Wang, X. Xi, C. A. Donnelly, A. C. Ghani, N. M. Ferguson, Estimates of the severity of coronavirus disease 2019: A model-based analysis. *Lancet Infect. Dis.* **20**, 669–677 (2020).
12. L. Fumanelli, M. Ajelli, P. Manfredi, A. Vespignani, S. Merler, Inferring the structure of social contacts from demographic data in the analysis of infectious diseases spread. *PLOS Comput. Biol.* **8**, e1002673 (2012).
13. N. Hens, G. M. Ayele, N. Goeyvaerts, M. Aerts, J. Mossong, J. W. Edmunds, P. Beutels, Estimating the impact of school closure on social mixing behaviour and the transmission of close contact infections in eight European countries. *BMC Infect. Dis.* **9**, 1–12 (2009).
14. N. Goeyvaerts, N. Hens, B. Ogunjimi, M. Aerts, Z. Shkedy, P. Van Damme, P. Beutels, Estimating infectious disease parameters from data on social contacts and serological status. *J. R. Stat. Soc. Ser. C Appl. Stat.* **59**, 255–277 (2010).
15. N. Goeyvaerts, E. Santermans, G. Potter, A. Torneri, K. Van Kerckhove, L. Willem, M. Aerts, P. Beutels, N. Hens, Household members do not contact each other at random: Implications for infectious disease modelling. *Proc. R. Soc. B* **285**, 20182201 (2018).
16. A. Aleta, D. Martín-Corral, M. A. Bakker, A. Pastore, Y. Piontti, M. Ajelli, M. Litvinova, M. Chinazzi, N. E. Dean, M. E. Halloran, I. M. Longini Jr., A. Pentland, A. Vespignani, Y. Moreno, E. Moro, Quantifying the importance and location of SARS-CoV-2 transmission events in large metropolitan areas. *Proc. Natl. Acad. Sci. U.S.A.* **119**, e2112182119 (2022).
17. C. Buckee, A. Noor, L. Sattenspiel, Thinking clearly about social aspects of infectious disease transmission. *Nature* **595**, 205–213 (2021).
18. M. Tizzoni, E. O. Nsoesie, L. Gauvin, M. Karsai, N. Perra, S. Bansal, Addressing the socioeconomic divide in computational modeling for infectious diseases. *Nat. Commun.* **13**, 1–7 (2022).
19. J. Bedson, L. A. Skrip, D. Pedi, S. Abramowitz, S. Carter, M. F. Jalloh, S. Funk, N. Gobat, T. Giles-Vernick, G. Chowell, J. Rangel, J. R. de Almeida, R. Elessawi, S. V. Scarpino, R. A. Hammond, S. Briand, J. M. Epstein, L. Hébert-Dufresne, B. M. Althouse, A review and agenda for integrated disease models including social and behavioural factors. *Nat. Hum. Behav.* **5**, 834–846 (2021).
20. J. Zelner, N. B. Masters, R. Naraharisetti, S. A. Mojola, M. Chowkwanyun, R. Malosh, There are no equal opportunity infectors: Epidemiological modelers must rethink our approach to inequality in infection risk. *PLOS Comput. Biol.* **18**, e1009795 (2022).
21. K. H. Grantz, M. S. Rane, H. Salje, G. E. Glass, S. E. Schachterle, D. A. Cummings, Disparities in influenza mortality and transmission related to sociodemographic factors within Chicago in the pandemic of 1918. *Proc. Natl. Acad. Sci. U.S.A.* **113**, 13839–13844 (2016).
22. S.-E. Mamelund, C. Shelley-Egan, O. Rogeberg, The association between socioeconomic status and pandemic influenza: Systematic review and meta-analysis. *PLOS ONE* **16**, e0244346 (2021).
23. K. A. Alexander, C. E. Sanderson, M. Marathe, B. L. Lewis, C. M. Rivers, J. Shaman, J. M. Drake, E. Lofgren, V. M. Dato, M. C. Eisenberg, S. Eubank, What factors might have led to the emergence of Ebola in West Africa? *PLOS Negl. Trop. Dis.* **9**, e0003652 (2015).
24. N. Perra, Non-pharmaceutical interventions during the COVID-19 pandemic: A review. *Phys. Rep.* **913**, 1–52 (2021).
25. R. Garnier, J. R. Benetka, J. Kraemer, S. Bansal, Socioeconomic disparities in social distancing during the COVID-19 pandemic in the United States: Observational study. *J. Med. Internet Res.* **23**, e24591 (2021).
26. W. Do Lee, M. Qian, T. Schwanen, The association between socioeconomic status and mobility reductions in the early stage of England's COVID-19 epidemic. *Health Place* **69**, 102563 (2021).
27. N. Gozzi, M. Tizzoni, M. Chinazzi, L. Ferres, A. Vespignani, N. Perra, Estimating the effect of social inequalities on the mitigation of COVID-19 across communities in Santiago de Chile. *Nat. Commun.* **12**, 1–9 (2021).
28. S. Heroy, I. Loaiza, A. Pentland, N. O'Clery, COVID-19 policy analysis: Labour structure dictates lockdown mobility behaviour. *J. R. Soc. Interface* **18**, 20201035 (2021).
29. L. Gauvin, P. Bajardi, E. Pepe, B. Lake, F. Privitera, M. Tizzoni, Socio-economic determinants of mobility responses during the first wave of COVID-19 in Italy: From provinces to neighbourhoods. *J. R. Soc. Interface* **18**, 20210092 (2021).
30. J. A. Weill, M. Stigler, O. Deschenes, M. R. Springborn, Social distancing responses to COVID-19 emergency declarations strongly differentiated by income. *Proc. Natl. Acad. Sci. U.S.A.* **117**, 19658–19660 (2020).
31. J. Jay, J. Bor, E. O. Nsoesie, S. K. Lipson, D. K. Jones, S. Galea, J. Raifman, Neighbourhood income and physical distancing during the COVID-19 pandemic in the United States. *Nat. Hum. Behav.* **4**, 1294–1302 (2020).
32. G. Bonaccorsi, F. Pierri, M. Cinelli, A. Flori, A. Galeazzi, F. Porcelli, A. L. Schmidt, C. M. Valensise, A. Scala, W. Quattrociocchi, F. Pammolli, Economic and social consequences of human mobility restrictions under COVID-19. *Proc. Natl. Acad. Sci. U.S.A.* **117**, 15530–15535 (2020).
33. S. J. Fox, E. Javan, R. Pasco, G. C. Gibson, B. Betke, J. L. Herrera-Diestra, S. Woody, K. Pierce, K. E. Johnson, M. Johnson-León, M. Lachmann, L. A. Meyers, Disproportionate impacts of COVID-19 in a large US city. *PLOS Comput. Biol.* **19**, e1011149 (2023).
34. N. Gozzi, M. Chinazzi, N. E. Dean, I. M. Longini Jr, M. Elizabeth Halloran, N. Perra, A. Vespignani, Estimating the impact of COVID-19 vaccine inequities: A modeling study. *Nat. Commun.* **14**, 3272 (2023).
35. A. Mousa, P. Winskill, O. J. Watson, O. Ratmann, M. Monod, M. Ajelli, A. Diallo, P. J. Dodd, C. G. Grijalva, M. C. Kiti, A. Krishnan, R. Kumar, S. Kumar, K. O. Kwok, C. F. Lanata, A. Melegaro, C. D. Morrow, J. Mossong, E. F. G. Neal, D. J. Nokes, W. Pan-ngum, G. E. Potter, F. M. Russell, S. Saha, J. D. Sugimoto, W. In Wei, R. R. Wood, J. T. Wu, J. Zhang, P. G. T. Walker, C. Whittaker, Social contact patterns and implications for infectious disease transmission–A systematic review and meta-analysis of contact surveys. *eLife* **10**, e70294 (2021).
36. O. Diekmann, J. A. Heesterbeek, M. G. Roberts, The construction of next-generation matrices for compartmental epidemic models. *J. R. Soc. Interface* **7**, 873–885 (2010).
37. C. B. Julie, L. M. Childs, An introduction to compartmental modeling for the budding infectious disease modeler. *Lett. Biomath.* **5**, 195–221 (2010).
38. P. Rohani M. Keeling, *Modeling Infectious Diseases in Humans and Animals* (Princeton Univ. Press, 2008).
39. A. Barrat, M. Barthelemy, A. Vespignani, *Dynamical Processes on Complex Networks* (Cambridge Univ. Press, 2012).
40. J. Koltai, O. Vásárhelyi, G. Röst, M. Karsai, Reconstructing social mixing patterns via weighted contact matrices from online and representative surveys. *Sci. Rep.* **12**, 1–12 (2022).
41. K. Sun, A. Baronchelli, N. Perra, Contrasting effects of strong ties on SIR and SIS processes in temporal networks. *Eur. Phys. J. B.* **88**, 1–8 (2015).
42. L. Di Domenico, G. Pullano, C. E. Sabbatini, P.-Y. Boëlle, V. Colizza, Impact of lockdown on COVID-19 epidemic in Île-de-France and possible exit strategies. *BMC Med.* **18**, 1–13 (2020).
43. Y. Leo, E. Fleury, J. I. Alvarez-Hamelin, C. Sarraute, M. Karsai, Socioeconomic correlations and stratification in social-communication networks. *J. R. Soc. Interface* **13**, 20160598 (2016).
44. X. Dong, A. J. Morales, E. Jahani, E. Moro, B. Lepri, B. Bozkaya, C. Sarraute, Y. Bar-Yam, A. Pentland, Segregated interactions in urban and online space. *EPJ Data Sci.* **9**, 7 (2020).
45. B. Oroszi, A. Juhász, C. Nagy, J. K. Horváth, K. E. Komlós, G. Túri, M. McKee, R. Ádány, Characteristics of the third COVID-19 pandemic wave with special focus on socioeconomic inequalities in morbidity, mortality and the uptake of COVID-19 vaccination in Hungary. *J. Pers. Med.* **12**, 388 (2022).

46. E. Valdano, J. Lee, S. Bansal, S. Rubrichi, V. Colizza, Highlighting socio-economic constraints on mobility reductions during COVID-19 restrictions in France can inform effective and equitable pandemic response. *J. Travel Med.* **28**, taab045 (2021).
47. M. Karmakar, P. M. Lantz, R. Tipirneni, Association of social and demographic factors with COVID-19 incidence and death rates in the US. *JAMA Netw. Open* **4**, e2036462 (2021).
48. J. Zelner, R. Trangucci, R. Naraharisetti, A. Cao, R. Malosh, K. Broen, N. Masters, P. Delamater, Racial disparities in coronavirus disease 2019 (COVID-19) mortality are driven by unequal infection risks. *Clin. Infect. Dis.* **72**, e88–e95 (2021).
49. J. Y.-M. Siu, Health inequality experienced by the socially disadvantaged populations during the outbreak of COVID-19 in Hong Kong: An interaction with social inequality. *Health Soc. Care Community* **29**, 1522–1529 (2021).
50. S. Sweeney, T. P. J. Capeding, R. Eggo, M. Huda, M. Jit, D. Mudzengi, N. R. Naylor, S. Procter, M. Quaife, L. Serebryakova, S. Torres-Rueda, V. Vargas; CHiL COVID Working Group, A. Vassall, Exploring equity in health and poverty impacts of control measures for SARS-CoV-2 in six countries. *BMJ Glob. Health* **6**, e005521 (2021).
51. K. C. Ma, T. F. Menkir, S. Kissler, Y. H. Grad, M. Lipsitch, Modeling the impact of racial and ethnic disparities on COVID-19 epidemic dynamics. *eLife* **10**, e66601 (2021).
52. S. Chang, E. Pierson, P. W. Koh, J. Gerardin, B. Redbird, D. Grusky, J. Leskovec, Mobility network models of COVID-19 explain inequities and inform reopening. *Nature* **589**, 82–87 (2021).
53. A. Aleta, D. Martin-Corral, A. Pastore, Y. Piontti, M. Ajelli, M. Litvinova, M. Chinazzi, N. E. Dean, M. E. Halloran, I. M. Longini Jr., S. Merler, A. Pentland, A. Vespignani, E. Moro, Y. Moreno, Modelling the impact of testing, contact tracing and household quarantine on second waves of COVID-19. *Nat. Hum. Behav.* **4**, 964–971 (2020).
54. M. Pangallo, A. Aleta, R. M. D. Rio-Chanona, A. Pichler, D. Martín-Corral, M. Chinazzi, F. Lafond, M. Ajelli, E. Moro, Y. Moreno, A. Vespignani, J. Doyne, Farmer, The unequal effects of the health–economy trade-off during the covid-19 pandemic. *Nat. Hum. Behav.* **8**, 264–275 (2024).
55. A. Machens, F. Gesualdo, C. Rizzo, A. E. Tozzi, A. Barrat, C. Cattuto, An infectious disease model on empirical networks of human contact: Bridging the gap between dynamic network data and contact matrices. *BMC Infect. Dis.* **13**, 185 (2013).
56. A. Aleta, G. F. de Arruda, Y. Moreno, Data-driven contact structures: From homogeneous mixing to multilayer networks. *PLOS Comput. Biol.* **16**, e1008035 (2020).
57. J. C. Miller, E. M. Volz, Incorporating disease and population structure into models of SIR disease in contact networks. *PLOS ONE* **8**, e69162 (2013).
58. M. Ajelli, B. Gonçalves, D. Balcan, V. Colizza, H. Hao, J. J. Ramasco, S. Merler, A. Vespignani, Comparing large-scale computational approaches to epidemic modeling: Agent-based versus structured metapopulation models. *BMC Infect. Dis.* **10**, 190 (2010).
59. P. Klepac, S. Kissler, J. Gog, Contagion! the BBC four pandemic–The model behind the documentary. *Epidemics* **24**, 49–59 (2018).
60. A. Melegaro, E. Del Fava, P. Poletti, S. Merler, C. Nyamukapa, J. Williams, S. Gregson, P. Manfredi, Social contact structures and time use patterns in the Manicaland Province of Zimbabwe. *PLOS ONE* **12**, e0170459 (2017).
61. M. Ajelli, M. Litvinova, Estimating contact patterns relevant to the spread of infectious diseases in Russia. *J. Theor. Biol.* **419**, 1–7 (2017).
62. J. Zhang, M. Litvinova, Y. Liang, Y. Wang, W. Wang, S. Zhao, W. Qianhui, S. Merler, C. Viboud, A. Vespignani, M. Ajelli, Y. Hongjie, Changes in contact patterns shape the dynamics of the COVID-19 outbreak in China. *Science* **368**, 1481–1486 (2020).
63. A. Gimma, J. D. Munday, K. L. M. Wong, P. Coletti, K. van Zandvoort, K. Prem, P. Klepac, G. J. Rubin, S. Funk, W. J. Edmunds, C. I. Jarvis; CMMID COVID-19 working group, Changes in social contacts in England during the COVID-19 pandemic between March 2020 and March 2021 as measured by the CoMix survey: A repeated cross-sectional study. *PLOS Med.* **19**, e1003907 (2022).
64. E. Moro, D. Calacci, X. Dong, A. Pentland, Mobility patterns are associated with experienced income segregation in large US cities. *Nat. Commun.* **12**, 4633 (2021).
65. T. Yabe, B. G. B. Bueno, X. Dong, A. Pentland, E. Moro, Behavioral changes during the COVID-19 pandemic decreased income diversity of urban encounters. *Nat. Commun.* **14**, 2310 (2023).
66. L. Rocher, J. M. Hendrickx, Y.-A. De Montjoye, Estimating the success of re-identifications in incomplete datasets using generative models. *Nat. Commun.* **10**, 3069 (2019).
67. N. Oliver, B. Lepri, H. Sterly, R. Lambiotte, S. Deletaille, M. De Nadai, E. Letouzé, A. A. Salah, R. Benjamins, C. Cattuto, V. Colizza, N. de Cordes, S. P. Fraiberger, T. Koebe, S. Lehmann, J. Murillo, A. Pentland, P. N. Pham, F. Pivetta, J. Saramäki, S. V. Scarpino, M. Tizzoni, S. Verhulst, P. Vinck, Mobile phone data for informing public health actions across the COVID-19 pandemic life cycle. *Sci. Adv.* **6**, eabc0764 (2020).
68. M. Karsai, J. Koltai, O. Vásárhelyi, G. Röst, Hungary in mask/maszk in Hungary. *Corvinus J. Sociol. Soc. Policy* **2**, 139–146 (2020).
69. C. D. Meyer, *Matrix Analysis and Applied Linear Algebra* (SIAM, 2023).
70. R. A. Horn, C. R. Johnson, *Matrix Analysis* (Cambridge Univ. Press, 2012.
71. Hungarian National Authority for Data Protection and Freedom of Information, *Nemzeti adatvédelmi és információ szabadság hatóság*, https://www.naih.hu/, 2023.
72. European Centre for Disease Prevention and Control, Surveillance definitions for COVID-19; www.ecdc.europa.eu/en/covid-19/surveillance/surveillance-definitions.
73. S. Arregui, A. Aleta, J. Sanz, Y. Moreno, Projecting social contact matrices to different demographic structures. *PLOS Comput. Biol.* **14**, e1006638 (2018).

Acknowledgments: We gratefully thank C. Cattuto and A. Vespignani for useful discussions. M.K. and A.M. are grateful to J. Koltai and Gergely Röst for their contribution in collecting and sharing the MASZK dataset. **Funding:** A.M. and M.K. were supported by the Accelnet-Multinet NSF grant. A.M. is grateful for the support from CEU and NetSI (Northeastern University). M.K. acknowledges support from the ANR project DATAREDUX (ANR-19-CE46–0008); the SoBigData++ H2020–871042; the EMOMAP CIVICA projects; and the National Laboratory for Health Security, Alfréd Rényi Institute, RRF-2.3.1-21-2022-00006. L.D. acknowledges support from the Fondation Botnar (EPFL COVID-19 Real Time Epidemiology I-DAIR Pathfinder) and from the Lagrange Project of the ISI Foundation funded by the CRT Foundation. **Author contributions:** A.M. performed the numerical simulations and data analysis. A.M., L.D., and N.P. developed the analytical formulation. A.M., M.T., and N.P. wrote the first draft of the manuscript. All authors designed the study, interpreted the results, and edited and approved the manuscript. **Competing interests:** The authors declare that they have no competing interests. **Data and materials availability:** All data needed to evaluate the conclusions in the paper are present in the paper and/or the Supplementary Materials. Python codes to reproduce the findings can be found here: https://doi.org/10.5281/zenodo.12533501.

Submitted 23 August 2023
Accepted 9 September 2024
Published 11 October 2024
10.1126/sciadv.adk4606
