# Supplementary material for: Generalized contact matrices allow integrating socioeconomic variables into epidemic models
Source: Sci Adv. 2024 Oct 11;10(41):eadk4606. doi: 10.1126/sciadv.adk4606 (PMC11468902; doi:10.1126/sciadv.adk4606)
Supplement: Supplementary file 2 — Supplementary Text Figs. S1 to S6 Tables S1 and S2 References [file sciadv.adk4606_sm.v2.pdf]

ERRATUM PUBLISHED 17 APRIL 2026; SEE BELOW

Science Advances
AAAS

# Supplementary Materials for

## Generalized contact matrices allow integrating socioeconomic variables into epidemic models

Adriana Manna *et al.*

Corresponding author: Nicola Perra, n.perra@qmul.ac.uk

*Sci. Adv.* **10**, eadk4606 (2024)
DOI: 10.1126/sciadv.adk4606

**This PDF file includes:**

Supplementary Text
Figs. S1 to S6
Tables S1 and S2
References

**Erratum (17 April 2026):** The original Supplementary Materials file contained discrepancies between the text and the insets of figs. S2, S4, S5. The main text stated the insets compared the three outcomes for a fixed value of $R_0$ ($R_0 = 2.7$). However, the authors had fixed the value of the transmissibility of the disease instead of $R_0$. Although the insets were correct, they did not correspond to what was written in the text. Edits have been made to the main text in the Results section to better reflect the relative position of peaks in the corrected figures. The *x*-axis range in Figs. 2, 3, and 4 in the main text and figs. S2, S4, S5, and S6 in the Supplementary Materials have been updated to make the plots more visible. The Supplementary Materials file has been replaced with the corrected versions of figs. S2, S4, S5, and S6. The authors' conclusions are not affected by these corrections.

# 1 Epidemic models with age-stratified contact matrices

Standard approaches to model the spreading of infectious diseases often acknowledge the stratification of contacts across age brackets. To this end, contact matrices $\mathbf{C}$ are introduced. The element $C_{ij}$ quantifies the average number of contacts that an individual in age-bracket $i$ has with individuals in age group $j$ within a certain time window [2, 3, 5]. The population is divided into age brackets so that $N = \sum_{i=1}^{K} N_i$. The variables $N_i$ capture the number of individuals in age group $i$ while $K$ indicates the number of different age groups. Given the definition of contact matrices, we can define $R_{ij} = C_{ij}N_i$ as the total number of contacts individuals in $i$ have with those in $j$. In matrix form, we can write $\mathbf{R} = \mathbf{NC}$, where $\mathbf{N}$ is diagonal and its elements describe the population in each age group. The $\mathbf{R}$ matrix is clearly symmetric: $R_{ji} = R_{ij}$. However, since in general $N_i \neq N_j$ the entries of the contact matrices are not.

Let us now consider a disease whose natural history can be described with a Susceptible-Exposed-Infected-Recovered model [38]. The epidemic dynamics are encoded in the following set of differential equations:

$$\begin{aligned} d_t S_i(t) &= -\Lambda_i(t) S_i(t), \\ d_t E_i(t) &= \Lambda_i(t) S_i(t) - \Psi E_i(t), \\ d_t I_i(t) &= \Psi E_i(t) - \Gamma I_i(t), \\ d_t R_i(t) &= \Gamma I_i(t). \end{aligned} \tag{S1}$$

Susceptible individuals, in contact with the infected, might be exposed to the virus with a rate driven by the force of infection $\Lambda_i(t)$; exposed are not yet infectious and transition to the infected compartment with rate $\Psi$; infected individuals recover with rate $\Gamma$. The force of infection is then defined as the per-capita rate at which susceptibles are exposed to the disease:

$$\Lambda_i(t) = \Phi \sum_{j=1}^{K} C_{ij} \frac{I_j(t)}{N_j} \tag{S2}$$

where $\Phi$ is the transmissibility of the disease and the temporal dependence is induced by the variation in the number of infected across age brackets.

## 1.1 Derivation of $R_0$

A fundamental quantity to understand the epidemic dynamics is the basic reproductive number, $R_0$, defined as the number of secondary infections generated by a single infected individual in an otherwise susceptible population [38]. The basic reproductive number is a function of the disease's features and the contact patterns of the population. The next-generation matrix approach can be used to determine a closed form expression for $R_0$. While we refer the reader to Ref. [37] for an overview of the method, in the following we provide a summary of its derivation. To keep equations more readable, we henceforth drop the explicit time dependence notation.

The first step is to focus our attention only on the compartments that describe any stages of the infection: $E_i$ and $I_i$ $i \in [1, K]$ in our case. It is convenient to re-write the differential equations for these compartments as

$$d_t \mathbf{x} = f(\mathbf{x}) - w(\mathbf{x}) \tag{S3}$$

where $\mathbf{x} \in [E_1, \dots E_K, I_1, \dots I_K]$, $f(\mathbf{x})$ encodes all terms that lead to infections and $w(\mathbf{x})$ all other transitions in and out of the compartments. More explicitly, we can write

$$d_t \mathbf{x} = \begin{bmatrix} d_t E_1 \\ \vdots \\ d_t E_K \\ d_t I_1 \\ \vdots \\ d_t I_K \end{bmatrix} = f(\mathbf{x}) - w(\mathbf{x}) = \begin{bmatrix} \Lambda_1 S_1 \\ \vdots \\ \Lambda_K S_K \\ 0 \\ \vdots \\ 0 \end{bmatrix} - \begin{bmatrix} \Psi E_1 \\ \vdots \\ \Psi E_K \\ \Gamma I_1 - \Psi E_1 \\ \vdots \\ \Gamma I_K - \Psi E_K \end{bmatrix} \tag{S4}$$

As alluded to above, the expression of $R_0$ is linked to the early epidemic dynamics which can be linearized by calculating the Jacobian at the disease-free equilibrium (DFE) $(S_i^*, E_i^*, I_i^*, R_i^*) = (N_i, 0, 0, 0)$ for all age groups. Using the Jacobian matrix $\mathbf{J}$ (which has size $2K \times 2K$) at the DFE we can write

$$d_t \mathbf{x} = \mathbf{J}\mathbf{x} \tag{S5}$$

This expression can be conveniently factorized as $d_t\mathbf{x} = (\mathbf{F} - \mathbf{W})\mathbf{x}$ where the matrix $\mathbf{F}$ is Jacobian applied to $f(\mathbf{x})$ and similarly the matrix $\mathbf{W}$ is the Jacobian applied to $w(\mathbf{x})$. In particular, we have:

$$\mathbf{F} = \begin{bmatrix} \partial_{E_1}(\Lambda_1 N_1) & \dots & \partial_{E_K}(\Lambda_1 N_1) & \partial_{I_1}(\Lambda_1 N_1) & \dots & \partial_{I_K}(\Lambda_1 N_1) \\ \partial_{E_1}(\Lambda_2 N_2) & \dots & \partial_{E_K}(\Lambda_2 N_2) & \partial_{I_1}(\Lambda_2 N_2) & \dots & \partial_{I_K}(\Lambda_2 N_2) \\ \vdots & \vdots & \vdots & \vdots & \vdots & \vdots \\ \partial_{E_1}(\Lambda_K N_K) & \dots & \partial_{E_K}(\Lambda_K N_K) & \partial_{I_1}(\Lambda_K N_K) & \dots & \partial_{I_K}(\Lambda_K N_K) \\ 0 & \dots & 0 & 0 & \dots & 0 \\ 0 & \dots & 0 & 0 & \dots & 0 \\ \vdots & \vdots & \vdots & \vdots & \vdots & \vdots \\ 0 & \dots & 0 & 0 & \dots & 0 \end{bmatrix} = \begin{bmatrix} 0 & \dots & 0 & \partial_{I_1}(\Lambda_1 N_1) & \dots & \partial_{I_K}(\Lambda_1 N_1) \\ 0 & \dots & 0 & \partial_{I_1}(\Lambda_2 N_2) & \dots & \partial_{I_K}(\Lambda_2 N_2) \\ \vdots & \vdots & \vdots & \vdots & \vdots & \vdots \\ 0 & \dots & 0 & \partial_{I_1}(\Lambda_K N_K) & \dots & \partial_{I_K}(\Lambda_K N_K) \\ 0 & \dots & 0 & 0 & \dots & 0 \\ 0 & \dots & 0 & 0 & \dots & 0 \\ \vdots & \vdots & \vdots & \vdots & \vdots & \vdots \\ 0 & \dots & 0 & 0 & \dots & 0 \end{bmatrix} \tag{S6}$$

We stress how the components of each gradient, are computed at the DFE, i.e., $S_i \to N_i$. By looking at Eq. S2 we note how each partial derivative in $p$ selects the $p$-th element of the sum hence:

$$\mathbf{F} = \Phi \begin{bmatrix} 0 & \dots & 0 & \frac{C_{11}}{N_1}N_1 & \dots & \frac{C_{1K}}{N_K}N_1 \\ 0 & \dots & 0 & \frac{C_{21}}{N_1}N_2 & \dots & \frac{C_{2K}}{N_K}N_2 \\ \vdots & \vdots & \vdots & \vdots & \vdots & \vdots \\ 0 & \dots & 0 & \frac{C_{K1}}{N_1}N_K & \dots & \frac{C_{KK}}{N_K}N_K \\ 0 & \dots & 0 & 0 & \dots & 0 \\ 0 & \dots & 0 & 0 & \dots & 0 \\ \vdots & \vdots & \vdots & \vdots & \vdots & \vdots \\ 0 & \dots & 0 & 0 & \dots & 0 \end{bmatrix} = \Phi \begin{bmatrix} \mathbf{0} & \tilde{\mathbf{C}} \\ \mathbf{0} & \mathbf{0} \end{bmatrix} \tag{S7}$$

where we denoted with $\mathbf{0}$ $K \times K$ blocks of zeros and the generic entry of $\tilde{\mathbf{C}}$ is $\tilde{C_{ij}} = \frac{C_{ij}}{N_j}N_i$. Using the same method we can easily write the expression of the matrix $\mathbf{W}$ as:

$$\mathbf{W} = \begin{bmatrix} \Psi & \dots & 0 & 0 & \dots & 0 \\ \vdots & \vdots & \vdots & \vdots & \vdots & \vdots \\ 0 & \dots & \Psi & 0 & \dots & 0 \\ -\Psi & \dots & 0 & \Gamma & \dots & 0 \\ \vdots & \vdots & \vdots & \vdots & \vdots & \vdots \\ 0 & \dots & -\Psi & 0 & \dots & \Gamma \end{bmatrix} = \begin{bmatrix} \Psi\mathbf{I} & \mathbf{0} \\ -\Psi\mathbf{I} & \Gamma\mathbf{I} \end{bmatrix} \tag{S8}$$

where $\mathbf{I}$ are identity matrices of size $K \times K$. The expression for $R_0$ is linked to the two matrices as $R_0 = \rho(\mathbf{F}\mathbf{W}^{-1})$, where $\rho$ denotes the spectral radius. It is easy to show how

$$\mathbf{F}\mathbf{W}^{-1} = \Phi \begin{bmatrix} \mathbf{0} & \tilde{\mathbf{C}} \\ \mathbf{0} & \mathbf{0} \end{bmatrix} \begin{bmatrix} \frac{1}{\Psi}\mathbf{I} & \mathbf{0} \\ \frac{1}{\Gamma}\mathbf{I} & \frac{1}{\Gamma}\mathbf{I} \end{bmatrix} = \frac{\Phi}{\Gamma} \begin{bmatrix} \tilde{\mathbf{C}} & \tilde{\mathbf{C}} \\ \mathbf{0} & \mathbf{0} \end{bmatrix} \tag{S9}$$

hence, we finally can write

$$R_0 = \frac{\Phi}{\Gamma}\rho(\tilde{\mathbf{C}}) \tag{S10}$$

Contact matrices are often stratified also for the context (i.e., location $l$) where interactions take place [5]. The entries $C_{ij}$ are then expressed as

$$C_{ij} = \sum_l \omega_l C_{ij}^{(l)} \tag{S11}$$

where $\omega_l$ are weights capturing possible heterogeneities in the relevance of contacts in each context in the transmission of the disease. In this formulation, the inclusion of the different contexts does not change the expression of $R_0$ as the $\omega_l$ are assumed to be homogeneous across age brackets.

## 2 Generalized contact matrices

In this work, we shift from the standard contact matrices that stratify contacts for age and possibly context, to a generalized version that allows for $m$ other dimensions. Here, we focus on socio-economic status (SES) variables as additional dimensions. However, the framework is general and can be applied to any categorical variable of epidemiological interest.

In more detail, we describe the generalized contact matrices as $G_{\mathbf{a},\mathbf{b}}$, where $\mathbf{a} = (i, \alpha, \beta, \dots, \gamma)$ and $\mathbf{b} = (j, \eta, \mu, \dots, \xi)$ are tuples (i.e., index vectors) representing individuals membership to each category. We note how we adopted Greek letters for the additional dimensions, though not strictly necessary. With these matrices we can, for example, capture contact stratification according to age, income ($\alpha$), and education attainment ($\beta$). In this scenario, $G_{\mathbf{a},\mathbf{b}}$ would describe the average number of contacts that an individual in age bracket $i$, income $\alpha$, and education $\beta$ has with people in age group $j$, income $\eta$, and education $\mu$ in a given time window. From this perspective, the matrix $\mathbf{C}$ can be thought of as an aggregation of contact patterns at a lower level of stratification. In other words, the standard matrices can be viewed as aggregations along all dimensions affecting the organization of contacts but age.
The total number of contacts of individuals in a given age group $i$ with others in $j$ can be written as $R_{ij} = C_{ij} N_i$. Considering the generalized contact matrices we have $C_{ij} N_i = \sum_{\mathbf{a}'\mathbf{b}'} G_{\mathbf{a}',\mathbf{b}'} N_{\mathbf{a}'}$, where $\mathbf{a}' = \mathbf{a} - \{i\}$ and $\mathbf{b}' = \mathbf{b} - \{j\}$ are the index vectors $m$ capturing all dimensions but age. Since the matrices $\mathbf{G}$ has $m+1$ dimensions, we can aggregate contacts in $m+1$ possible ways, for example computing the average contacts that an individual in one of the SES $\alpha$ has with others in the same SES $\beta$ obtaining

$$C_{\alpha\beta} N_\alpha = \sum_{\mathbf{a}'\mathbf{b}'} G_{\mathbf{a}',\mathbf{b}'} N_{\mathbf{a}'} \tag{S12}$$

where now $\mathbf{a}' = \mathbf{a} - \{\alpha\}$ and $\mathbf{b}' = \mathbf{b} - \{\beta\}$. In general, $C_{ij} \neq C_{\alpha\beta}$. Indeed, the number of groups and the number of individuals in each of them might be different. Looking at contact patterns from multiple dimensions of stratification allows us to observe the way interactions are aggregated (i.e., by age or other dimensions) might affect the estimation of key epidemiological parameters.

We denote with $K$ the number of age groups, while with $V_p$ the number of groups in each $m$ other dimensions (i.e., $p \in [1, m]$). While the generalized matrix $\mathbf{G}$ can be naturally described as a multidimensional matrix, the use of $T = K \prod_{p=1}^{m} V_p$ index vectors pairs allows for flattened representation in a squared bi-dimensional matrix of size $T \times T$. We note how the formulation can easily consider different contexts where interactions take places, i.e., $G_{\mathbf{a},\mathbf{b}} = \sum_l \omega_l G^{(l)}_{\mathbf{a},\mathbf{b}}$ where $\omega_l$ captures the relative importance of the different social settings in the transmission [5].

## 2.1 Synthetic generalized contact matrices

In this section, we provide details about the generation of synthetic generalized contact matrices we built to explore the possible effects of different mixing patterns among individuals. For simplicity, we first consider only two dimensions: age and a socio-economic status (SES) variable (though any other categorical variable could be used).

We developed a model to derive $G_{\mathbf{a},\mathbf{b}}$ where $\mathbf{a} = (i, \alpha)$ and $\mathbf{b} = (j, \beta)$, and $i$ and $j$ refers to the age group while $\alpha$ and $\beta$ to the SES of the ego and the alter respectively. We start from an empirical contact matrix $\mathbf{C}$ describing the contact rates between age brackets $i$ and $j$. As mentioned above, we can define $\mathbf{R} = \mathbf{NC}$ as the total number of contacts between the two groups in the given period. While the matrix $C_{ij}$ is not symmetric, $R_{ij}$ is since the number of *raw* contacts between two groups is the same, i.e., $R_{ij} = C_{ij} N_i = R_{ji} = C_{ji} N_j$. To build the generalized contact matrices $G_{\mathbf{a},\mathbf{b}}$ we first split the total contacts $R_{ij}$ across the second dimension, and then we compute the contacts rates. In other words $R_{ij} = \sum_{\alpha,\beta} (R_G)_{i\alpha,j\beta}$. The problem is then how to get values for the elements $(R_G)_{i\alpha,j\beta}$. It is useful to reflect on the properties of these matrices. First, $(R_G)_{i\alpha,j\beta} = (R_G)_{j\beta,i\alpha}$. In other words, the number of contacts that individuals in age group $i$ and SES $\alpha$ have with individuals in age group $j$ and SES $\beta$ must equal the number of contacts that individuals in age group $j$ and SES $\beta$ have with individuals in age group $i$ and SES $\alpha$. This property implies that $(R_G)_{i\alpha,j\beta} = (R_G)^{\top}_{i\alpha,j\beta}$. Indeed, for a given pair of indices $i$ and $j$, one can think about $(R_G)_{i\alpha,j\beta}$ as a $V_1 \times V_1$ matrix (since $\alpha, \beta \in [1, \dots, V_1]$), which describes the contact patterns between those two age groups across SES. For all $i = j$ the symmetry of matrix $(R_G)_{i\alpha,i\beta}$ implies $(R_G)_{i\alpha,i\beta} = (R_G)_{i\beta,i\alpha}$. In general this is not the case for $i \neq j$. Indeed, the overall symmetry of the matrix it is not enough to guarantee that $(R_G)_{i\alpha,j\beta} = (R_G)_{i\beta,j\alpha}$ (note how just the indices of the second dimension have been swapped). This would imply a much stronger internal symmetry for each pair $i$ and $j$ which is not generally required. Second, as mentioned above the sum over all $\alpha$ and $\beta$ is set by the number of contacts between age group $i$ and $j$: $R_{ij} = \sum_{\alpha,\beta} (R_G)_{i\alpha,j\beta}$.

Given these features, how can we set the entries of these matrices? As mentioned, for a given pair $i$ and $j$ the matrix $(R_G)_{i\alpha,j\beta}$ is of size $V_1 \times V_1$ since $\alpha, \beta \in [1, \dots, V_1]$. For any $i \neq j$, we need a model to set $W = V_1^2 - 1$ elements of the matrix (the minus one is due to the constraint introduced by the second

property defined above). For all $i = j$ instead, the symmetry of the matrix is such that we need to set only $Y = V_1 + \frac{V_1(V_1-1)}{2} - 1$ elements. The first $V_1$ comes from the diagonal (i.e., $\alpha = \beta$), the $\frac{V_1(V_1-1)}{2}$ are instead the off-diagonal values, the minus one is due to the constraint of the total number of contacts which is set by $R_{ij}$. Since $W > Y$ for all $V_1 \geq 2$, we can define the matrix by defining $W$ values. In doing so, we assume an independence between age and SES. To set the $W$ values we consider that for any $i$ and $j$ pair:

1. each SES $\alpha$ is generally responsible for $P_\alpha$ fraction of $R_{ij}$ connections, where $\sum_\alpha P_\alpha = 1$
2. we assume that a fraction $q_\alpha$ of these are on the diagonal (i.e., in-group connections) and $1 - q_\alpha$ are instead off-diagonal. This parameter controls the assortativity of connections within each group.

In what follows, we consider the fractions $P_\alpha$ and $q_\alpha$ as input parameters. In case $W$ is equal to this number $(2V_1 - 1)$, the constraints imposed by our assumptions allow to define all the entries of the matrix. If instead $W > 2V_1 - 1$, other $W - 2V_1 + 1$ parameters are required.

Let us consider first the case in which $V_1 = 3$, $\alpha, \beta \in [1, 2, 3]$. In these settings $W = 8$, and $Y = 5$ which is equal to $2V_1 - 1$. Hence, defining the two set of input parameters is enough to obtain the matrix for any $i = j$, while still 3 additional parameters are needed for $i \neq j$. It is important to stress how the first property described above implies that we need to define only this number for all $i < j$. The correspondent values for $i > j$ are readily obtained just by transposing the matrices. It is useful, to split the two cases:

**Case $i = j$.**

For all $i = j$ the matrices $(R_G)_{i\alpha,i\beta}$ take the form of

$$(R_G)_{i\alpha,i\beta} = R_{ii} \begin{pmatrix} q_1 P_1 & p_{i1,i2} & p_{i1,i3} \\ p_{i1,i2} & q_2 P_2 & p_{i2,i3} \\ p_{i1,i3} & p_{i2,i3} & q_3 P_3 \end{pmatrix} \tag{S13}$$

where each $p_{i\alpha,i\beta} \in [0, 1]$ is defined such that $(R_G)_{i\alpha,i\beta} = R_{ii} p_{i\alpha,i\beta}$. As the matrix needs to be symmetric we have written, for example, $p_{i1,i2}$ instead of $p_{i2,i1}$. Due to our assumptions, the sum of the off-diagonal elements of each row should sum to $(1 - q_\alpha)P_\alpha = \Pi_\alpha$, while on the diagonal we have $q_\alpha P_\alpha$. Thus the following system of equations must be respected:

$$\begin{cases} p_{i1,i2} + p_{i1,i3} = \Pi_1 \\ p_{i1,i2} + p_{i2,i3} = \Pi_2 \\ p_{i1,i3} + p_{i2,i3} = \Pi_3 \end{cases} \tag{S14}$$

The solution, if any, is unique and can be written as

$$\hat{\mathbf{p}} = \mathbf{A}^{-1}\mathbf{\Pi} = \frac{1}{2} \begin{pmatrix} \Pi_1 + \Pi_2 - \Pi_3 \\ \Pi_1 + \Pi_3 - \Pi_2 \\ \Pi_2 + \Pi_3 - \Pi_1 \end{pmatrix} \tag{S15}$$

where

$$\mathbf{A} = \begin{pmatrix} 1 & 1 & 0 \\ 1 & 0 & 1 \\ 0 & 1 & 1 \end{pmatrix}, \quad \mathbf{\Pi} = \begin{pmatrix} \Pi_1 \\ \Pi_2 \\ \Pi_3 \end{pmatrix}, \quad \hat{\mathbf{p}} = \begin{pmatrix} p_{i1,i2} \\ p_{i1,i3} \\ p_{i2,i3} \end{pmatrix} \tag{S16}$$

Let us consider now the case in which $V_1 = 4$. In this case, $Y = 9$. As before, $2V_1 - 1$ parameters are inputs (three $P_\alpha$ and four $q_\alpha$). In this case, $Y > 2V_1 - 1$. Hence, the system of $V_1$ differential equations in $V_1(V_1 - 1)/2 = 6$ (off-diagonal) elements requires $Y - 2V_1 + 1 = 2$ free parameters. Indeed, the matrix $(R_G)_{i\alpha,i\beta}$ can be written as:

$$(R_G)_{i\alpha,i\beta} = R_{ii} \begin{pmatrix} q_1 P_1 & p_{i1,i2} & p_{i1,i3} & p_{i1,i4} \\ p_{i1,i2} & q_2 P_2 & p_{i2,i3} & p_{i2,i4} \\ p_{i1,i3} & p_{i2,i3} & q_3 P_3 & p_{i3,i4} \\ p_{i1,i4} & p_{i2,i4} & p_{i3,i4} & q_4 P_4 \end{pmatrix} \tag{S17}$$

Considering that the sum of each off-diagonal element in each row $\alpha$ should sum to $(1 - q_\alpha)P_\alpha$ we obtain a system of four equations:

$$\begin{cases} p_{i1,i2} + p_{i1,i3} + p_{i1,i4} = \Pi_1 \\ p_{i1,i2} + p_{i2,i3} + p_{i2,i4} = \Pi_2 \\ p_{i1,i3} + p_{i2,i3} + p_{i3,i4} = \Pi_3 \\ p_{i1,i4} + p_{i2,i4} + p_{i3,i4} = \Pi_4 \end{cases} \tag{S18}$$

As before, we can write the system of 6 equations in matrix form as $\mathbf{A}\hat{\mathbf{p}} = \mathbf{\Pi}$ where:

$$\mathbf{A} = \begin{pmatrix} 1 & 1 & 1 & 0 & 0 & 0 \\ 1 & 0 & 0 & 1 & 1 & 0 \\ 0 & 1 & 0 & 1 & 0 & 1 \\ 0 & 0 & 1 & 0 & 1 & 1 \end{pmatrix}, \hat{\mathbf{p}} = \begin{pmatrix} p_{i1,i2} \\ p_{i1,i3} \\ p_{i1,i4} \\ p_{i2,i3} \\ p_{i2,i4} \\ p_{i3,i4} \end{pmatrix}, \mathbf{\Pi} = \begin{pmatrix} \Pi_1 \\ \Pi_2 \\ \Pi_3 \\ \Pi_4 \end{pmatrix} \tag{S19}$$

Clearly the system is under-determined as it has four equations and six variables. To find the solution we need to fix two of such variables, for example, $p_{i2,i4}$ and $p_{i3,i4}$. Doing so, we can write the system of equations as $\mathbf{A_r}\hat{\mathbf{p}}_\mathbf{r} = \mathbf{\Pi_r}$ where

$$\mathbf{A}_r = \begin{pmatrix} 1 & 1 & 1 & 0 \\ 1 & 0 & 0 & 1 \\ 0 & 1 & 0 & 1 \\ 0 & 0 & 1 & 0 \end{pmatrix}, \hat{\mathbf{p}}_\mathbf{r} = \begin{pmatrix} p_{i1,i2} \\ p_{i1,i3} \\ p_{i1,i4} \\ p_{i2,i3} \end{pmatrix}, \mathbf{\Pi_r} = \begin{pmatrix} \Pi_1 \\ \Pi_2 - p_{i2,i4} \\ \Pi_3 - p_{i3,i4} \\ \Pi_4 - p_{i2,i4} - p_{i3,i4} \end{pmatrix} \tag{S20}$$

The subscript $r$ stands for reduced, as by fixing the $Y - 2V_1 + 1$ variables we can move from a $4 \times 6$ matrix to a $4 \times 4$. The solution, if any, is now unique and can be written as:

$$\hat{\mathbf{p}}_\mathbf{r} = \mathbf{A_r^{-1}}\mathbf{\Pi_r} \tag{S21}$$

For any general $V_1$, the method described above allows to define the matrix $(R_G)_{i\alpha,i\beta}$ passing through an under-defined system of linear equations. By setting the potential $Y - 2V_1 + 1$ free parameters the solution, if it exists, is unique. However, we expect that some solutions might not be physical. Indeed, the matrix entries must be all equal or larger than zero. Any solution that leads to negative values is not physical and must be neglected. This condition adds constraints to the possible values of the $Y$ parameters. It is important to stress how the free parameters are in general functions of $i$. For simplicity, and to avoid the need to set values across all age groups, we set them equal for all age groups.

**Case $i < j$.**

For all $i < j$ the matrices $(R_G)_{i\alpha,j\beta}$ take the form of

$$(R_G)_{i\alpha,j\beta} = R_{ij} \begin{pmatrix} q_1 P_1 & p_{i1,j2} & p_{i1,j3} \\ p_{i2,j1} & q_2 P_2 & p_{i2,j3} \\ p_{i3,j1} & p_{i3,j2} & q_3 P_3 \end{pmatrix} \tag{S22}$$

where each $p_{i\alpha,j\beta} \in [0,1]$ is defined such that $(R_G)_{i\alpha,j\beta} = R_{ij} p_{i\alpha,j\beta}$. The difference with respect to the previous case is that, since $i \neq j$, we have $p_{i\alpha,j\beta} \neq p_{i\beta,j\alpha}$. Hence, the matrix $\mathbf{p}$ is not symmetric for swaps of indices in the second dimension. As such, we need to find a model to set the $W = V_1^2 - 1$ values. However, as in the previous case, due to our assumptions, the sum of the off-diagonal elements of each row should sum to $(1 - q_\alpha)P_\alpha = \Pi_\alpha$, while on the diagonal we have $q_\alpha P_\alpha$. Thus the following system of equations must be respected:

$$\begin{cases} p_{i1,j2} + p_{i1,j3} = \Pi_1 \\ p_{i2,j1} + p_{i2,j3} = \Pi_2 \\ p_{i3,j1} + p_{i3,j2} = \Pi_3 \end{cases} \tag{S23}$$

The system of equations is clearly under-defined. Hence, even for the scenario with $V_1 = 3$ we have three parameters (one per equation). For example, we could set as parameters $p_{i1,j2}$, $p_{i2,j1}$, and $p_{i3,j1}$. This choice leads to the solution

$$\begin{cases} p_{i1,j3} = \Pi_1 - p_{i1,j2} \\ p_{i2,j3} = \Pi_2 - p_{i2,j1} \\ p_{i3,j2} = \Pi_3 - p_{i3,j1} \end{cases} \tag{S24}$$

Since the values of all the entries are defined between 0 and 1, each parameter is bounded by the following conditions: $0 \leq p_{i1,j2} \leq \Pi_1$, $0 \leq p_{i2,j1} \leq \Pi_2$, and $0 \leq p_{i3,j1} \leq \Pi_3$. Considering these constraints and the simple structure of Eq. S24 for $V_1 = 3$, we can set the three free parameters as $p_{i1,j2} = a_1\Pi_1$, $p_{i2,j1} = a_2\Pi_2$, and $p_{i3,j1} = a_3\Pi_3$ where $a_1$, $a_2$, and $a_3$ are unknowns defined between zero and one. The use of $a_i$ with $i = [1, 2, 3]$, though not necessary, allows for a more physical interpretation of the free parameters as a fraction of the off-diagonal contacts. It is important to stress how once we have picked these three parameters, the matrix $(R_G)_{i\alpha,j\beta}$ is fully defined as well as the matrix $(R_G)_{j\beta,i\alpha}$ which is the transpose. Furthermore, we have three parameters for each $i < j$, since the entries are in principle functions of the age groups. However, for simplicity we set the same three free parameters across all age combinations.

Let us consider now the case in which $V_1 = 4$. In this case, $W = V_1^2 - 1 = 15$. As before, $2V_1 - 1$ parameters are inputs (three $P_\alpha$ and four $q_\alpha$). In this case, $W > 2V_1 - 1$. Hence, the system of $V_1$ differential equations in $V_1(V_1 - 1)$ (off-diagonal) elements required $W - 2V_1 + 1 = 8$ free parameters. We note how in this case, and more in general for any $V_1 > 3$, we cannot simply adopt, without introducing further assumptions, $a_i$ values as we did above for $V_1 = 3$. The most general solution can be obtained by defining directly some of the entries of the matrix (i.e., free parameters) and solving the resulting system of equations.

## 2.2 Random mixing regime

By setting $P_\alpha = q_\alpha = N_\alpha/N$ (where $N_\alpha = \sum_i N_{i\alpha}$) the model drastically simplifies and the contacts division of the total contact $R_{ij}$ between age groups $i$ and $j$ across $\alpha$ and $\beta$ equal the product of the fraction of these two groups (independently of age). This is the random mixing regime, indeed, if contacts are random across groups the expectation value of this process leads to the aforementioned product. To provide a concrete example, let us consider a simple case where we have only one SES dimension and $V_1 = 3$. As described above, for all $i < j$ in these settings $W = 8$ and

$$(R_G)_{i\alpha,j\beta} = R_{ij} \begin{pmatrix} q_1 P_1 & p_{i1,j2} & p_{i1,j3} \\ p_{i2,j1} & q_2 P_2 & p_{i2,j3} \\ p_{i3,j1} & p_{i3,j2} & q_3 P_3 \end{pmatrix} \tag{S25}$$

Now we can set the parameters in the diagonal equal to the fraction of the population of each group:

$$(R_G)_{i\alpha,j\beta} = R_{ij} \begin{pmatrix} \frac{N_1}{N}\frac{N_1}{N} & p_{i1,j2} & p_{i1,j3} \\ p_{i2,j1} & \frac{N_2}{N}\frac{N_2}{N} & p_{i2,j3} \\ p_{i3,j1} & p_{i3,j2} & \frac{N_3}{N}\frac{N_3}{N} \end{pmatrix} \tag{S26}$$

The off-diagonal values are defined similarly

$$(R_G)_{i\alpha,j\beta} = R_{ij} \begin{pmatrix} \frac{N_1^2}{N^2} & \frac{N_1 N_2}{N^2} & \frac{N_1 N_3}{N^2} \\ \frac{N_1 N_2}{N^2} & \frac{N_2^2}{N^2} & \frac{N_2 N_3}{N^2} \\ \frac{N_1 N_3}{N^2} & \frac{N_2 N_3}{N^2} & \frac{N_3^2}{N^2} \end{pmatrix} \tag{S27}$$

The assumption that the division of the $R_{ij}$ contacts for SES depends only on the total fraction of individuals in SES induces symmetry in these matrices even for the case $i < j$. We note how, independently of the number of SES groups, assigning contacts at random proportionally to the populations allows us to define matrices without the need to set free parameters. It is easy to show that by setting $P_\alpha = q_\alpha = N_\alpha/N$, the matrices are defined in such a way that the on-diagonal elements have a $P_\alpha q_\alpha$ fraction of contacts while the off-diagonal elements in each row sum to $P_\alpha(1 - q_\alpha) = \Pi_\alpha$

Finally, we note how the more general way to define a homogeneous mixing scenario is done considering $(R_G)_{i\alpha,j\beta} = R_{ij}\frac{N_{i\alpha}}{N_i}\frac{N_{j\beta}}{N_j}$. In this case, we split the total contacts considering the fraction of individuals in each age and SES group. In doing so, the potential dependence of age is fully considered and the homogeneous mixing is defined for each pair of age groups. This is the general case we have considered in the corollary of the theorem described in details below. It is important to notice that this model does not, in general, lead to the symmetry of the $\mathbf{p}$ matrices for $i < j$. However, it reduces to the previous assuming that $\frac{N_{i\alpha}}{N_i} = \frac{N_\alpha}{N}$ for $\forall i$, hence when the fraction of individuals in each SES is independent of age.

# 3 Epidemic models with generalized contact matrices

In this section, we provide a detailed description of epidemic models featuring generalized contact matrices. As done above, let us consider a disease whose natural history can be described with a Susceptible-Exposed-Infected-Recovered model [38]. By considering the population sliced in $m+1$ dimensions the epidemic dynamics are encoded in the following set of differential equations:

$$\begin{aligned} d_t S_{\mathbf{a}}(t) &= -\Lambda_{\mathbf{a}}(t) S_{\mathbf{a}}(t), \\ d_t E_{\mathbf{a}}(t) &= \Lambda_{\mathbf{a}}(t) S_{\mathbf{a}}(t) - \Psi E_{\mathbf{a}}(t), \\ d_t I_{\mathbf{a}}(t) &= \Psi E_{\mathbf{a}}(t) - \Gamma I_{\mathbf{a}}(t), \\ d_t R_{\mathbf{a}}(t) &= \Gamma I_{\mathbf{a}}(t). \end{aligned} \tag{S28}$$

where $\mathbf{a} = (i, \alpha, \ldots, \gamma)$ is the index vector describing the membership of individuals in the $m+1$ groups. In other words, the unfolding of the disease is captured by $4 \times K \prod_{p=1}^{m} V_p$ compartments. The force of infection, defined as the per-capita rate at which susceptible are exposed to the disease, can be written as:

$$\Lambda_{\mathbf{a}}(t) = \Phi \sum_{\mathbf{b}} G_{\mathbf{a},\mathbf{b}} \frac{I_{\mathbf{b}}(t)}{N_{\mathbf{b}}} \tag{S29}$$

where $\Phi$ is the transmissibility of the disease and the temporal dependence is induced by the variation in the number of infected across age brackets.

## Derivation of $R_0$

The derivation of the basic reproductive number is done following the next generation matrix approach [37]. As done for standard contact matrices above, the first step is to focus only on the compartments that describe any stages of the infection: $E_{\mathbf{a}}$ and $I_{\mathbf{a}}$. It is convenient to re-write the differential equations for these compartments as

$$d_t \mathbf{x} = f(\mathbf{x}) - w(\mathbf{x}) \tag{S30}$$

where $\mathbf{x} \in [E_{\mathbf{1}}, \ldots E_{\mathbf{T}}, I_{\mathbf{1}}, \ldots I_{\mathbf{T}}]$, $f(\mathbf{x})$ encodes all terms that lead to infections and $w(\mathbf{x})$ all other transitions in and out of the compartments. Furthermore, we denote with $\mathbf{a}$ ($a \in [1, T]$ and $T = K \prod_{p=1}^{m} V_p$) the index vectors. In other words, $\mathbf{1}$ describes the index vector $(1, 1, \ldots, 1)$ of size $m+1$, $\mathbf{2}$ the index vector $(1, 1, \ldots, 2)$, and $\mathbf{T}$ instead $(K, V_1, \ldots, V_m)$. Using this notation, we map the generalized problem to the same structure described above for only one dimension (i.e., age). Indeed, we can write, once again dropping the time dependence for convenience,

$$d_t \mathbf{x} = \begin{bmatrix} d_t E_{\mathbf{1}} \\ \vdots \\ d_t E_{\mathbf{T}} \\ d_t I_{\mathbf{1}} \\ \vdots \\ d_t I_{\mathbf{T}} \end{bmatrix} = f(\mathbf{x}) - w(\mathbf{x}) = \begin{bmatrix} \Lambda_{\mathbf{1}} S_{\mathbf{1}} \\ \vdots \\ \Lambda_{\mathbf{T}} S_{\mathbf{T}} \\ 0 \\ \vdots \\ 0 \end{bmatrix} - \begin{bmatrix} \Psi E_{\mathbf{1}} \\ \vdots \\ \Psi E_{\mathbf{T}} \\ \Gamma I_{\mathbf{1}} - \Psi E_{\mathbf{1}} \\ \vdots \\ \Gamma I_{\mathbf{T}} - \Psi E_{\mathbf{T}} \end{bmatrix} \tag{S31}$$

Which has exactly the same structure as the analogous described above, but where $i \to \mathbf{a}$ and $\mathbf{a} = (i, \alpha_1, \ldots, \alpha_m)$. As shown above, the expression of $R_0$ is linked to the early epidemic dynamics which can be linearized by calculating the Jacobian at the disease-free equilibrium (DFE) $(S^*_{\mathbf{j}}, E^*_{\mathbf{j}}, I^*_{\mathbf{j}}, R^*_{\mathbf{j}}) = (N_{\mathbf{j}}, 0, 0, 0)$ for all age groups. Using the Jacobian matrix $\mathbf{J}$ (which has size $2K \prod_{p=1}^{m} V_p \times 2K \prod_{p=1}^{m} V_p$) at the DFE we can write

$$d_t \mathbf{x} = \mathbf{J} \mathbf{x} \tag{S32}$$

This expression can be conveniently factorized as $d_t\mathbf{x} = (\mathbf{F} - \mathbf{W})\mathbf{x}$ where the matrix $\mathbf{F}$ is Jacobian applied to $f(\mathbf{x})$ and similarly the matrix $\mathbf{W}$ is the Jacobian applied to $w(\mathbf{x})$. In particular, we have:

$$
\mathbf{F} = \begin{bmatrix}
\partial_{E_{\mathbf{1}}}(\Lambda_{\mathbf{1}}N_{\mathbf{1}}) & \dots & \partial_{E_{\mathbf{T}}}(\Lambda_{\mathbf{1}}N_{\mathbf{1}}) & \partial_{I_{\mathbf{1}}}(\Lambda_{\mathbf{1}}N_{\mathbf{1}}) & \dots & \partial_{I_{\mathbf{T}}}(\Lambda_{\mathbf{1}}N_{\mathbf{1}}) \\
\partial_{E_{\mathbf{1}}}(\Lambda_{\mathbf{2}}N_{\mathbf{2}}) & \dots & \partial_{E_{\mathbf{T}}}(\Lambda_{\mathbf{2}}N_{\mathbf{2}}) & \partial_{I_{\mathbf{1}}}(\Lambda_{\mathbf{2}}N_{\mathbf{2}}) & \dots & \partial_{I_{\mathbf{T}}}(\Lambda_{\mathbf{2}}N_{\mathbf{2}}) \\
\vdots & \vdots & \vdots & \vdots & \vdots & \vdots \\
\partial_{E_{\mathbf{1}}}(\Lambda_{\mathbf{T}}N_{\mathbf{T}}) & \dots & \partial_{E_{\mathbf{T}}}(\Lambda_{\mathbf{T}}N_{\mathbf{T}}) & \partial_{I_{\mathbf{1}}}(\Lambda_{\mathbf{T}}N_{\mathbf{T}}) & \dots & \partial_{I_{\mathbf{T}}}(\Lambda_{\mathbf{T}}N_{\mathbf{T}}) \\
0 & \dots & 0 & 0 & \dots & 0 \\
0 & \dots & 0 & 0 & \dots & 0 \\
\vdots & \vdots & \vdots & \vdots & \vdots & \vdots \\
0 & \dots & 0 & 0 & \dots & 0
\end{bmatrix} = \begin{bmatrix}
0 & \dots & 0 & \partial_{I_{\mathbf{1}}}(\Lambda_{\mathbf{1}}N_{\mathbf{1}}) & \dots & \partial_{I_{\mathbf{T}}}(\Lambda_{\mathbf{1}}N_{\mathbf{1}}) \\
0 & \dots & 0 & \partial_{I_{\mathbf{1}}}(\Lambda_{\mathbf{2}}N_{\mathbf{2}}) & \dots & \partial_{I_{\mathbf{T}}}(\Lambda_{\mathbf{2}}N_{\mathbf{2}}) \\
\vdots & \vdots & \vdots & \vdots & \vdots & \vdots \\
0 & \dots & 0 & \partial_{I_{\mathbf{1}}}(\Lambda_{\mathbf{T}}N_{\mathbf{T}}) & \dots & \partial_{I_{\mathbf{T}}}(\Lambda_{\mathbf{T}}N_{\mathbf{T}}) \\
0 & \dots & 0 & 0 & \dots & 0 \\
0 & \dots & 0 & 0 & \dots & 0 \\
\vdots & \vdots & \vdots & \vdots & \vdots & \vdots \\
0 & \dots & 0 & 0 & \dots & 0
\end{bmatrix} \tag{S33}
$$

The components of each gradient are computed at the DFE, i.e., $S_{\mathbf{j}} \to N_{\mathbf{j}}$. By looking at Eq. S29 we note how each partial derivative in $\mathbf{j}$ selects the $j$-th index vector:

$$
\mathbf{F} = \Phi \begin{bmatrix}
0 & \dots & 0 & \frac{G_{\mathbf{1},\mathbf{1}}}{N_{\mathbf{1}}}N_{\mathbf{1}} & \dots & \frac{G_{\mathbf{1},\mathbf{T}}}{N_{\mathbf{T}}}N_{\mathbf{1}} \\
0 & \dots & 0 & \frac{G_{\mathbf{2},\mathbf{1}}}{N_{\mathbf{1}}}N_{\mathbf{2}} & \dots & \frac{G_{\mathbf{2},\mathbf{T}}}{N_{\mathbf{T}}}N_{\mathbf{2}} \\
\vdots & \vdots & \vdots & \vdots & \vdots & \vdots \\
0 & \dots & 0 & \frac{G_{\mathbf{K},\mathbf{1}}}{N_{\mathbf{1}}}N_{\mathbf{T}} & \dots & \frac{G_{\mathbf{T},\mathbf{T}}}{N_{\mathbf{T}}}N_{\mathbf{T}} \\
0 & \dots & 0 & 0 & \dots & 0 \\
0 & \dots & 0 & 0 & \dots & 0 \\
\vdots & \vdots & \vdots & \vdots & \vdots & \vdots \\
0 & \dots & 0 & 0 & \dots & 0
\end{bmatrix} = \Phi \begin{bmatrix} \mathbf{0} & \tilde{\mathbf{G}} \\ \mathbf{0} & \mathbf{0} \end{bmatrix} \tag{S34}
$$

where we denoted with $\mathbf{0}$ $K\prod_{p=1}^{m} V_p \times K\prod_{p=1}^{m} V_p$ blocks of zeros and the generic entry of $\tilde{\mathbf{G}}$ is $\tilde{G}_{\mathbf{a},\mathbf{b}} = \frac{G_{\mathbf{a},\mathbf{b}}}{N_{\mathbf{b}}}N_{\mathbf{a}}$. Using the same method we can easily write the expression of the matrix $\mathbf{V_1}$ as:

$$
\mathbf{W} = \begin{bmatrix}
\Psi & \dots & 0 & 0 & \dots & 0 & & \\
\vdots & \vdots & \vdots & \vdots & \vdots & \vdots & \vdots & \vdots \\
0 & \dots & \Psi & 0 & \dots & 0 & & \\
-\Psi & \dots & 0 & \Gamma & \dots & 0 & & \\
\vdots & \vdots & \vdots & \vdots & \vdots & \vdots & \vdots & \vdots \\
0 & \dots & -\Psi & 0 & \dots & \Gamma & &
\end{bmatrix} = \begin{bmatrix} \Psi\mathbf{I} & \mathbf{0} \\ -\Psi\mathbf{I} & \Gamma\mathbf{I} \end{bmatrix} \tag{S35}
$$

where $\mathbf{I}$ are diagonal matrices of size $K\prod_{p=1}^{m} V_p \times K\prod_{p=1}^{m} V_p$. The expression for $R_0$ is linked to the two matrices as $R_0 = \rho(\mathbf{F}\mathbf{W}^{-1})$, where $\rho$ denotes the spectral radius. It is easy to show how

$$
\mathbf{F}\mathbf{W}^{-1} = \Phi \begin{bmatrix} \mathbf{0} & \tilde{\mathbf{G}} \\ \mathbf{0} & \mathbf{0} \end{bmatrix} \begin{bmatrix} \frac{1}{\Psi}\mathbf{I} & \mathbf{0} \\ \frac{1}{\Gamma}\mathbf{I} & \frac{1}{\Gamma}\mathbf{I} \end{bmatrix} = \frac{\Phi}{\Gamma} \begin{bmatrix} \tilde{\mathbf{G}} & \tilde{\mathbf{G}} \\ \mathbf{0} & \mathbf{0} \end{bmatrix} \tag{S36}
$$

hence, we can finally write

$$
R_0 = \frac{\Phi}{\Gamma}\rho(\tilde{\mathbf{G}}) \tag{S37}
$$

The expression has the same general form as the one derived for standard contact matrices stratified just for age. However, the entries of the matrix are different and those encode the multiple dimensions. Contact matrices are often stratified also for the context (location) where they take place [5]. The entries $G_{\mathbf{a};\mathbf{b}}$ are then expressed as

$$
G_{\mathbf{a},\mathbf{b}} = \sum_l \omega_l G^{(l)}_{\mathbf{a},\mathbf{b}} \tag{S38}
$$

where $\omega_s$ are weights that capture possible heterogeneities in the relevance of contacts in each context in the disease transmission. In this formulation, the inclusion of the different contexts does not change the expression of $R_0$ as the $\omega_l$ are assumed to be homogeneous across age brackets.

# 4 On the spectral radius of generalized contact matrices

We provide some formal relations between the spectral radius of the generalized contact matrices and the classical ones. We denote $\tilde{\mathbf{C}}$ in matrix form as $\tilde{\mathbf{C}} = \mathbf{R}\mathbf{N}^{-1}$, where $\mathbf{N}$ is diagonal with positive elements

and describes the number of individuals in each age group, $\mathbf{R}$ is symmetric and capturing the number of contacts between pairs. We can similarly denote the generalized contact matrix $\tilde{\mathbf{G}} = \mathbf{R_G}\mathbf{N_G}^{-1}$, where $\mathbf{N_G}$ is now the diagonal matrix of the generalized populations sizeswith positive elements. We note how the requirement that the diagonal matrices featuring the population sizes have positive elements implies that all sub-groups are not empty. Technically, it is a requirement needed to ensure the existence of their inverse. In the following, unless specified otherwise, we consider the simple case in which the generalized contact matrices feature two dimensions, that is, one additional dimension besides age. As we stated already, these two contact matrices have clearly different sizes that we denote as $\tilde{\mathbf{C}} \in \mathbb{R}^{K\times K}$ and $\tilde{\mathbf{G}} \in \mathbb{R}^{KV_1\times KV_1}$. To move from $\tilde{\mathbf{C}}$ to $\tilde{\mathbf{G}}$ we thus need an "inflation" process that turns every element of $\tilde{\mathbf{C}}$ into a matrix of size $V_1 \times V_1$. To lighten the notation, in the remainder of this section, we use $V$ instead of $V_1$.

## 4.1 Theorem enunciation

**Theorem 1** (Spectral radius of generalized contact matrices)**.** *Let $\tilde{\mathbf{C}} = \mathbf{R}\mathbf{N}^{-1} \in \mathbb{R}^{K\times K}$ be a single-attribute contact matrix where $\mathbf{R}$ is a symmetric, non-negative matrix, and $\mathbf{N}$ is a diagonal matrix, with positive diagonal elements. Let $\tilde{\mathbf{G}} = \mathbf{R_G}\mathbf{N_G}^{-1} \in \mathbb{R}^{KV\times KV}$ be a two-attributes generalization of $\tilde{\mathbf{C}}$ defined such that $\sum_{\alpha,\beta=1}^{V}(R_G)_{i\alpha,j\beta} = R_{ij}$ and $\sum_{\alpha,\beta=1}^{V}(N_G)_{i\alpha,j\beta} = N_{ij}$ with $\mathbf{R_G}$ being a symmetric, non-negative matrix, and $\mathbf{N_G}$ being a diagonal matrix with positive diagonal elements. By denoting with $\rho(\mathbf{M})$ the spectral radius of a generic matrix $\mathbf{M}$ it follows $\rho(\tilde{\mathbf{G}}) \geq \rho(\tilde{\mathbf{C}})$.*

From the theorem, the following corollary follows:

**Corollary 1.** *Under the same assumptions of Theorem 1, denote with $\hat{N} \in \mathbb{R}^K$ and $\hat{N}_G \in \mathbb{R}^{KV}$ the diagonal of $\mathbf{N}, \mathbf{N_G}$, respectively. If $\mathbf{R}$ is irreducible and*

$$(\mathbf{R_G})_{i\alpha,j\beta} = \mathbf{R}_{ij} \cdot \frac{\hat{N}_{\mathbf{G},i\alpha}\hat{N}_{\mathbf{G},j\beta}}{\hat{N}_i\hat{N}_j},$$

*then $\rho(\tilde{\mathbf{G}}) = \rho(\tilde{\mathbf{C}})$.*

In simple words, this theorem states that a generalized contact matrix always has a greater or equal spectral radius (hence reproductive number $R_0$) than its standard counterpart. The corollary focuses on the case in which the generalized contact matrix is built under a random mixing assumption, i.e., assuming that the number of contacts between $(i\alpha, j\beta)$ can be derived from $R_{ij}$ simply assuming that they are proportional to the product between the group sizes of $(i\alpha), (j\beta)$. The corollary asserts that, in this case, the spectral radius does not change and thus highlights the importance of measuring the *actual* contact matrix and not deriving it from a simple homogeneous mixing hypothesis. The technical assumption that $\mathbf{R}$ is irreducible implies that the contact matrix can be represented as a connected graph. Indeed, if the non-generalized contact matrix is not irreducible it means that there are sets of age groups that never interact together. This perfect segregation scenario is a corner case that is best described by running different epidemic models on each connected component. Consequently, Corollary 1 still holds under very general conditions.

## 4.2 Proof

We want to relate the spectral radius of the contact matrix $\tilde{\mathbf{C}}$ to its generalized counterpart $\tilde{\mathbf{G}}$. Both matrices are non-negative and real, but in general not symmetric.

The key steps of the proof are the following. The first step is defining a symmetric version of the two contact matrices with the same eigenvalues. This allows us to leverage several properties of real symmetric matrices. The second step is proving Corollary 1 gathering useful insights about these matrices in the case of homogeneous mixing. The third step is to go back to the general case and leverage the Rayleigh-Ritz theorem which links the leading eigenvalue of a symmetric real matrix with its so-called Rayleigh quotient. Finally, we use the quotient to how the main claim of the theorem holds.

### 4.2.1 Definition of symmetrized contact matrices

Following the notation introduced above, we define the following two matrices:

$$\tilde{\mathbf{C}}^{\text{sym}} = \mathbf{N}^{-1/2}\mathbf{R}\mathbf{N}^{-1/2} \tag{S39}$$

$$\tilde{\mathbf{G}}^{\text{sym}} = \mathbf{N_G}^{-1/2}\mathbf{R_G}\mathbf{N_G}^{-1/2}. \tag{S40}$$

These matrices are indeed symmetric because

$$\tilde{\mathbf{C}}^{\text{sym}}_{ij} = \frac{\mathbf{R}_{ij}}{\sqrt{\hat{N}_i \hat{N}_j}} = \frac{\mathbf{R}_{ji}}{\sqrt{\hat{N}_i \hat{N}_j}} = \tilde{\mathbf{C}}^{\text{sym}}_{ji}.$$

The relation above follows from $\mathbf{R}$ being symmetric and can be equivalently obtained for $\tilde{\mathbf{G}}^{\text{sym}}$. We show that all the eigenvalues of $\tilde{\mathbf{C}}$ and $\tilde{\mathbf{G}}$ are eigenvalues of $\tilde{\mathbf{C}}^{\text{sym}}$ and $\tilde{\mathbf{G}}^{\text{sym}}$. For simplicity, we now only show this for $\tilde{\mathbf{C}}$, but the result follows for $\tilde{\mathbf{G}}$ after a change of notation. Let $\mathbf{x}$ be an eigenvector of $\tilde{\mathbf{C}}$ with eigenvalue $\gamma$, then $\mathbf{y} = \mathbf{N}^{-1/2}\mathbf{x}$ is an (un-normalized) eigenvector of $\tilde{\mathbf{C}}^{\text{sym}}$ with eigenvalue $\gamma$. Indeed:

$$\begin{aligned} \tilde{\mathbf{C}}\mathbf{x} &= \gamma\mathbf{x} \\ \mathbf{R}\mathbf{N}^{-1}\mathbf{x} &\overset{(a)}{=} \gamma\mathbf{x} \\ \mathbf{R}\mathbf{N}^{-1}\underbrace{\mathbf{N}^{1/2}\mathbf{y}}_{\mathbf{x}} &\overset{(b)}{=} \gamma\underbrace{\mathbf{N}^{1/2}\mathbf{y}}_{\mathbf{x}} \\ \mathbf{N}^{-1/2}\mathbf{R}\mathbf{N}^{-1/2}\mathbf{y} &\overset{(c)}{=} \gamma\mathbf{y} \\ \tilde{\mathbf{C}}^{\text{sym}}\mathbf{y} &\overset{(d)}{=} \gamma\mathbf{y}, \end{aligned} \tag{S41}$$

proving the statement. In $(a)$ we used the definition $\tilde{\mathbf{C}} = \mathbf{R}\mathbf{N}^{-1}$; in $(b)$ we used $\mathbf{y} = \mathbf{N}^{-1/2}\mathbf{x}$; in $(c)$ we multiplied on both sides by $\mathbf{N}^{1/2}$; finally in $(d)$ we introduced $\tilde{\mathbf{C}}^{\text{sym}}$. Since this relation holds for all eigenvectors of $\tilde{\mathbf{C}}$, it follows that the two matrices are similar, i.e., they have the same eigenvalues. Then, proving that $\rho(\tilde{\mathbf{G}}^{\text{sym}}) \geq \rho(\tilde{\mathbf{C}}^{\text{sym}})$ is equivalent to proving $\rho(\tilde{\mathbf{G}}) \geq \rho(\tilde{\mathbf{C}})$. In other words, the following relation holds in general:

$$\rho(\tilde{\mathbf{G}}^{\text{sym}}) \geq \rho(\tilde{\mathbf{C}}^{\text{sym}}) \iff \rho(\tilde{\mathbf{G}}) \geq \rho(\tilde{\mathbf{C}}).$$

allowing us to work with the symmetric version of the matrices in the remainder. We now proceed first to prove the equality enunciated in Corollary 1 and then prove Theorem 1.

### 4.2.2 Spectral radius for homogeneous mixing

To prove the equality we use a *guess eigenvector* strategy, i.e., we provide an explicit expression of the leading eigenvector[1] of $\tilde{\mathbf{G}}^{\text{sym}}$ as a function of the leading eigenvector of $\tilde{\mathbf{C}}$ and show that is an exact eigenvector. Recall that we are considering the case in which $\mathbf{R_G}$ has the form:

$$(\mathbf{R_G})_{i\alpha,j\beta} = \mathbf{R}_{ij} \cdot \frac{\hat{N}_{G,i\alpha}\hat{N}_{G,j\beta}}{\hat{N}_i \hat{N}_j}. \tag{S42}$$

Note that from this definition, if $\mathbf{R}$ is irreducible, then $\mathbf{R}_G$ is irreducible as well as the matrices $\tilde{\mathbf{C}}^{\text{sym}}$, and $\tilde{\mathbf{G}}^{\text{sym}}$. In these settings, we start from the following relations which are verified under the homogeneous mixing assumption:

$$\begin{aligned} \tilde{\mathbf{G}}^{\text{sym}}_{i\alpha,j\beta} &\overset{(e)}{=} \hat{N}^{-1/2}_{\mathbf{G},i\alpha}\,(\mathbf{R_G})_{i\alpha,j\beta}\,\hat{N}^{-1/2}_{\mathbf{G},j\beta} \overset{(f)}{=} \frac{\hat{N}^{1/2}_{\mathbf{G},i\alpha}}{\hat{N}_i} \cdot \mathbf{R}_{ij} \cdot \frac{\hat{N}^{1/2}_{\mathbf{G},j\beta}}{\hat{N}_j} \\ &\overset{(g)}{=} \frac{\hat{N}^{1/2}_{\mathbf{G},i\alpha}}{\hat{N}_i} \cdot \underbrace{\hat{N}^{1/2}_i \tilde{\mathbf{C}}^{\text{sym}}_{ij} \hat{N}^{1/2}_j}_{\mathbf{R}_{ij}} \cdot \frac{\hat{N}^{1/2}_{\mathbf{G},j\beta}}{\hat{N}_j} = \sqrt{\frac{\hat{N}_{G,i\alpha}\hat{N}_{G,j\beta}}{\hat{N}_i \hat{N}_j}} \cdot \tilde{\mathbf{C}}^{\text{sym}}_{ij}, \end{aligned}$$

where $(e)$ follows from the definition given in Equation (S40); $(f)$ follows from the definition given in Equation (S42), and $(g)$ follows from Equation (S39).

Let $\mathbf{x} \in \mathbb{R}^K$ be the leading eigenvector of $\tilde{\mathbf{C}}^{\text{sym}}$ and let $\rho$ be its associated eigenvalue. We define $\mathbf{y} \in \mathbb{R}^{KV}$ as $\mathbf{y}_{i\alpha} = \sqrt{\frac{\hat{N}_{G,i\alpha}}{\hat{N}_i}} \cdot \mathbf{x}_i$. We can show that $\mathbf{y}$ is the (un-normalized) leading eigenvector of $\tilde{\mathbf{G}}^{\text{sym}}$

[1]The leading eigenvector is the eigenvector associated with the largest eigenvalue.

with eigenvalue $\rho$, in fact

$$\left(\tilde{\mathbf{G}}^{\text{sym}}\mathbf{y}\right)_{i\alpha} = \sum_{j\beta} \tilde{\mathbf{G}}^{\text{sym}}_{i\alpha,j\beta} \cdot \mathbf{y}_{j\beta} = \sum_{j\beta} \underbrace{\sqrt{\frac{\hat{N}_{G,i\alpha}\hat{N}_{G,j\beta}}{\hat{N}_i\hat{N}_j}} \cdot \tilde{\mathbf{C}}^{\text{sym}}_{ij}}_{\tilde{\mathbf{G}}^{\text{sym}}_{i\alpha,j\beta}} \cdot \underbrace{\sqrt{\frac{\hat{N}_{G,j\beta}}{\hat{N}_j}} \cdot \mathbf{x}_j}_{\mathbf{y}_{j\beta}}$$

$$= \sqrt{\frac{\hat{N}_{G,i\alpha}}{\hat{N}_i}} \cdot \sum_j \frac{\tilde{\mathbf{C}}^{\text{sym}}_{ij}}{\hat{N}_j} \cdot \mathbf{x}_j \cdot \sum_\beta \hat{N}_{G,j\beta} \overset{(h)}{=} \sqrt{\frac{\hat{N}_{G,i\alpha}}{\hat{N}_i}} \cdot \sum_j \tilde{\mathbf{C}}^{\text{sym}}_{ij}\mathbf{x}_j$$

$$\overset{(i)}{=} \sqrt{\frac{\hat{N}_{G,i\alpha}}{\hat{N}_i}} \cdot \rho \mathbf{x}_i \overset{(l)}{=} \rho \cdot \mathbf{y}_{i\alpha},$$

where in $(h)$ we exploited the relation $\hat{N}_i = \sum_\alpha \hat{N}_{G,i\alpha}$, in $(i)$ we used $\tilde{\mathbf{C}}^{\text{sym}}\mathbf{x} = \rho\mathbf{x}$ and in $(l)$ we used the definition of $\mathbf{y}$. This result tells us that $\rho$, the spectral radius of $\tilde{\mathbf{C}}^{\text{sym}}$ is also an eigenvalue of $\tilde{\mathbf{G}}^{\text{sym}}$. Now, to conclude the proof, we exploit Perron-Frobenius theorem (see for instance [69], Chapter 8) stating that for a non-negative irreducible matrix (such as $\tilde{\mathbf{G}}^{\text{sym}}$), the leading eigenvector is the only positive eigenvector. Since $\tilde{\mathbf{C}}^{\text{sym}}$ is non-negative, then it follows that its leading eigenvector $\mathbf{x}$ is also positive. This however implies that $\mathbf{y}$ is positive and we thus obtain that $\rho$ is the spectral radius of $\tilde{\mathbf{G}}^{\text{sym}}$, concluding the proof.

### 4.2.3 Spectral radius for arbitrary generalized contact matrix

We now provide the proof of the main theorem. From the Rayleigh-Ritz theorem [70, Theorem 4.2.2], we know that for a symmetric real matrix, the leading eigenvalue (or spectral radius) satisfies the relation:

$$\rho(\tilde{\mathbf{G}}^{\text{sym}}) = \max_{\mathbf{z}\in\mathbb{R}^{VK}\neq\mathbf{0}} \mathcal{R}(\tilde{\mathbf{G}}^{\text{sym}}, \mathbf{z}),$$

where

$$\mathcal{R}(\tilde{\mathbf{G}}, \mathbf{z}) = \frac{\mathbf{z}^T\tilde{\mathbf{G}}^{\text{sym}}\mathbf{z}}{\mathbf{z}^T\mathbf{z}}$$

is called the Rayleigh quotient. This allows one to find the inequality $\rho(\tilde{\mathbf{G}}^{\text{sym}}) \geq \mathcal{R}(\tilde{\mathbf{G}}^{\text{sym}}, \mathbf{y})$ for all $\mathbf{y}$. We are thus left to find $\mathbf{y}$ so that $\mathcal{R}(\tilde{\mathbf{G}}^{\text{sym}}, \mathbf{y}) = \rho(\tilde{\mathbf{C}}^{\text{sym}})$ to conclude the proof. Consider the vector $\mathbf{y} \in \mathbb{R}^{KV}$ so that $\mathbf{y}_{i\alpha} = \sqrt{\frac{\hat{N}_{G,i\alpha}}{\hat{N}_i}} \cdot \mathbf{x}_i$, as it was earlier defined. Recalling that

$$\tilde{\mathbf{G}}^{\text{sym}}_{i\alpha,j\beta} = \frac{(\mathbf{R}_\mathbf{G})_{i\alpha,j\beta}}{\sqrt{\hat{N}_{G,i\alpha}\hat{N}_{G,j\beta}}},$$

we can write

$$\rho\left(\tilde{\mathbf{G}}^{\text{sym}}\right) = \max_{\mathbf{z}\in\mathbb{R}^{VK}\neq\mathbf{0}} \frac{\mathbf{z}^T\tilde{\mathbf{G}}^{\text{sym}}\mathbf{z}}{\mathbf{z}^T\mathbf{z}} \geq \frac{\mathbf{y}^T\tilde{\mathbf{G}}^{\text{sym}}\mathbf{y}}{\mathbf{y}^T\mathbf{y}} = \frac{\sum_{i\alpha}\sum_{j\beta}\sqrt{\frac{\hat{N}_{G,i\alpha}\hat{N}_{G,j\beta}}{\hat{N}_i\hat{N}_j}}\frac{(\mathbf{R}_\mathbf{G})_{i\alpha,j\beta}}{\sqrt{\hat{N}_{G,i\alpha}\hat{N}_{G,j\beta}}}\mathbf{x}_i\mathbf{x}_j}{\sum_{i\alpha}\frac{\hat{N}_{G,i\alpha}}{\hat{N}_i}\mathbf{x}_i^2}$$

$$= \frac{\sum_{ij}\frac{\mathbf{x}_i\mathbf{x}_j}{\sqrt{\hat{N}_i\hat{N}_j}}\sum_{\alpha\beta}(\mathbf{R}_\mathbf{G})_{i\alpha,j\beta}}{\sum_i\frac{\mathbf{x}_i^2}{\hat{N}_i}\sum_\alpha\hat{N}_{G,i\alpha}} \overset{(m)}{=} \frac{\sum_{ij}\frac{\mathbf{x}_i\mathbf{x}_j}{\sqrt{\hat{N}_i\hat{N}_j}}\mathbf{R}_{ij}}{\sum_i\frac{\mathbf{x}_i^2}{\hat{N}_i}\hat{N}_i} = \frac{\sum_{ij}\tilde{\mathbf{C}}^{\text{sym}}_{ij}\mathbf{x}_i\mathbf{x}_j}{\|\mathbf{x}\|^2} = \rho,$$

where in $(m)$ we exploited the relation $\sum_{\alpha,\beta}(\mathbf{R}_\mathbf{G})_{i\alpha,j\beta} = \mathbf{R}_{ij}$ and that $\hat{N}_i = \sum_\alpha \hat{N}_{G,i\alpha}$. Thus we obtain $\rho\left(\tilde{\mathbf{G}}^{\text{sym}}\right) \geq \rho$, concluding the proof.

### 4.2.4 Extension to generalized matrices with more than two dimensions

Our theorem relates the spectral radius of a single-attribute contact matrix $\tilde{\mathbf{C}}$ with its generalized version, obtained by adding a single additional attribute (i.e., dimension). In the main text, we claimed that the same relation proven above holds also for multiple attributes additions. The matrix $\mathbf{R}$ is of size $K \times K$,

while its generalized counterpart $\mathbf{R_G}$ (used in the theorem) is of size $KV_1 \times KV_1$. The crucial observation is that the proof relies on the flattened representation of the generalized contact matrix and does not make any assumption on the number of dimensions actually added. Hence, it works in general for any number of dimensions, provided that the relation detailed in Theorem1 between $\mathbf{R}$ and $\mathbf{R_G}$ holds.

Given this premise, let us now consider a generalized contact matrix with $r = m + 1$ attributes $\tilde{\mathbf{G}}_r$ and size $T_r = K \prod_{p=1}^{m} V_p$ and another generalized version obtained adding $l$ further attributes, denoted with $\tilde{\mathbf{G}}_{r+l}$ and size $T_{r+l} = K \prod_{p=1}^{m+l} V_p$. The matrix $\tilde{\mathbf{G}}_r$ is mathematically equivalent to a single attribute matrix in which each attribute can take $T_r$ different values (instead of just $K$). We now add one attribute to it and, from Theorem 1, we obtain that $\rho(\tilde{\mathbf{G}}_{r+1}) \geq \rho(\tilde{\mathbf{G}}_r)$. Adding iteratively one attribute at the time we obtain

$$\rho(\tilde{\mathbf{G}}_{r+l}) \geq \rho(\tilde{\mathbf{G}}_{r+l-1}) \geq \cdots \geq \rho(\tilde{\mathbf{G}}_{r+1}) \geq \rho(\tilde{\mathbf{G}}_r),$$

thus showing that any additional attribute cannot decrease the spectral radius.

# 5 Data

## 5.1 The MASZK questionnaire

In this study we used data coming from the MASZK survey study [40, 68], a large data collection effort on social mixing patterns made during the COVID-19 pandemic, conducted in Hungary from April 2020 to July 2022. The study involved 26 monthly cross-sectional anonymous phone surveys using Computer Assisted Telephone Interview (CATI) methodology, with a nationally representative sample of 1000 participants each month. The recorded population was representative in terms of gender, age, education level, and type of settlement. Sampling errors were further corrected by post-stratification weights. The data collection adhered to European and Hungarian privacy regulations, approved by the Hungarian National Authority for Data Protection and Freedom of Information [71], as well as the Health Science Council Scientific and Research Ethics Committee (resolution number IV/3073-1/2021/EKU).

Relevant to this study, the questionnaires recorded information about the *proxy social contacts*, defined as interactions where the respondent and a peer stayed within 2 meters for more than 15 minutes [72], at least one of them without wearing a mask. Approximate contact numbers were recorded between the respondents and their peers from different age groups of 0–4, 5–14, 15–29, 30–44, 45–59, 60–69, 70–79, and 80+. Contact number data about underage children were collected by asking legal guardians to estimate daily contact patterns. Participants during the whole data collection were asked to report contacts referring ($i$) to the previous day (that we use for the analysis in Fig 4 in the main text) and, during the first data collection campaigns ($ii$) to an average pre-pandemic day (that we use for the analysis in Fig 2 and 3 in the main text). Additionally, in three data collection waves: April 2021, November 2021, and June 2022 contacts have been collected in the form of diaries. Namely, participants were asked to list one by one the contacts they had on the previous day by providing some socio-demographic information about the contacts such as their wealth situation. Beyond information on contacts before and during the pandemic, the MASZK dataset provided us with information on *social-demographic characteristics* of participants, such as their *perceived wealth situation*, *gender*, *vaccination status*, etc.

### 5.1.1 Other contacts data

Beyond the data on Hungary, we use data on Zimbabwe age-contact patterns [60]. Also, in this case, the contact numbers among age classes have been collected through surveys.

### 5.1.2 Age-contact matrices

Although contacts (i.e., raw contacts), theoretically, are expected to be symmetrical, due to survey limitations and reporting errors they do not achieve perfect reciprocity. Given that our model to build synthetic generalized contact matrices respects the symmetric assumption on the raw contacts, we needed to ensure that also the matrix $\mathbf{R}$ that we fed to these models respects this assumption as well. Thus, all the raw contact matrices used in this study have been corrected for reciprocity *a priori*. This consists of averaging the total number of contacts measured in one direction, from $i$ to $j$, and the reciprocal from $j$ to $i$ (Eq S43) [73].

$$C_{ij} = \frac{C_{ij}^{data} N_i + C_{ji}^{data} N_j}{2N_i} = \frac{R_{ij}}{N_i} \tag{S43}$$

Where, $N_i$ indicates the number of participants in age class $i$ in each study, and $C_{ij}^{data} N_i$ is the total number of contacts reported in the survey from individuals in age class $i$ with individuals in age class $j$.

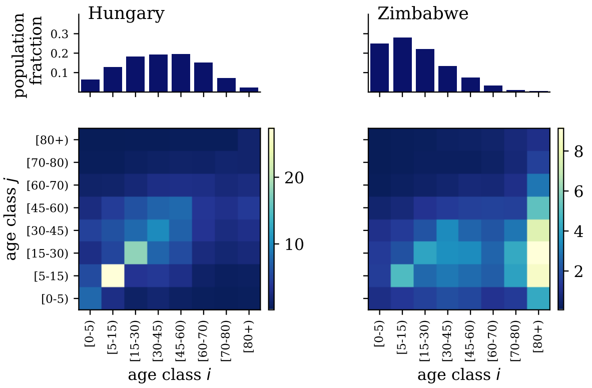


Fig. S1: **Empirical age distrubutions and age structured contact matrices.** Population distribution by age ($1st$ *row*) and age-structured contact matrices ($2nd$ *row*) referred to the pre-pandemic period for Hungary and Zimbabwe.

# 6 Parameters and implementation

To ensure the reproducibility of our work, in this section we include a comprehensive list of all the parameters employed to generate the synthetic and empirical generalized contact matrices used for the analysis presented in the main text. In addition, we provide all the technical details about the implementation of the analysis.

## Figure 2

In Table S1 we report the list of parameters used to generate the generalized contact matrices for the analysis presented in Figure 2 in the main text. For the random mixing scenario the only set of parameters of interest is the population distribution. Indeed, in these settings, no extra parameters are needed. While, for the assortative regime we also report the values of the activity and assortativity. As described above, for $V_1 = 3$, in general, we need to fix $W = V_1^2 - 1 = 8$ free parameters. $2V_1 - 1 = 5$ are defined by the assumption of our model (i.e., by setting $P_\alpha$ and $q_\alpha$ values). Hence, three extra values are required to fully define the generalized contact matrix. We set these values as $a_1 = 0.6$ (correspondent to $p_{i1,j2} = 0.048$ in Eq. S24), $a_2 = 0.6$ (correspondent to $p_{i2,j1} = 0.12$ in Eq. S24), and $a_3 = 0.5$ (correspondent to $p_{i3,j1} = 0.07$ in Eq. S24) respectively for the first, second and third SES group. We refer the reader to Section 2.1 for the details about the synthetic generalized contact matrices construction.

| SES Group | **1** | **2** | **3** |
|---|---|---|---|
| Population distribution | 35% | 45% | 20% |
| Assortativity ($q_i$) | 60% | 50% | 65% |
| Activity ($P_i$) | 20% | 40% | 40% |
| Free parameters ($a_i$) | 0.6 | 0.6 | 0.5 |

Table S1: List of parameters used to generate Figure 2 in the main text.

## Figure 3

To demonstrate the flexibility of the proposed approach to model NPIs in Figure 3 we analyze three different scenarios: ($i$) Baseline, ($ii$) NPI 1 and ($iii$) NPI 2. In particular, in the scenario of NPI

1, we assume that all the individuals reduce their contacts by 35% at $t^* = 50$, while in the scenario of NPI 2, we assume that the introduction of NPI also affects the structure of the matrix. Thus, to model these scenarios, we first reduce by 35% the total number of contacts across age groups such that $R_{ij}^{NPI} = 0.65 R_{ij}$. Then, we feed $R_{ij}^{NPI}$ to our model to generate synthetic generalized contact matrices. For the first scenario (NPI 1), the parameters of the model are equal to one of the baseline scenarios, while, the parameters of the second scenario (NPI 2) change according to the assumption that we made on the structure of contacts. In Table S2 we report the parameters used to generate the generalized contact matrices for this analysis. As for the previous figure, three additional free parameters are needed to define the generalized contact matrix. These are, for the baseline, $a_1 = 0.5$, $a_2 = 0.5$, $a_3 = 0.5$ and $a_1 = 0.5$, $a_2 = 0.4$, $a_3 = 0.4$ for NPI2.

| | **Baseline** | | | **NPI 2** | | |
|---|---|---|---|---|---|---|
| SES Group | **1** | **2** | **3** | **1** | **2** | **3** |
| Population distribution | 33,3% | 33,3% | 33,3% | 33,3% | 33,3% | 33,3% |
| Assortativity ($q_i$) | 33,3% | 50% | 60% | 50% | 60% | 70% |
| Activity ($P_i$) | 25% | 45% | 30% | 37% | 37% | 26% |
| Free parameters ($a_i$) | 0.5 | 0.5 | 0.5 | 0.5 | 0.4 | 0.4 |

Table S2: List of parameters used to generate Figure 3 in the main text, for ($i$) baseline and, ($ii$) NPI 2 scenarios.

### Figure 4

We built generalized contact matrices stratified in two dimensions by using real data from the *MASZK* study.

Information on social interactions has been collected in two different ways i) in an aggregate form, such that each participant reported the number of contacts they had with individuals in each of the eight age brackets considered, ii) in a diary in which each participant listed one by one the interactions they had on a given day by reporting some meta information of the *contactee* such as their age and SES. In particular, the average number of contacts of an individual in age class $i$, and SES $\alpha$ with an individual in age class $j$, and SES $\beta$ is $G_{\mathbf{a},\mathbf{b}}$ where $\mathbf{a} = (i, \alpha)$ and $\mathbf{b} = (j, \beta)$. However, the *MASZK* data provided us with diary information only for the adult population (individuals older than 15 years old).

For what concerns children, the *MASZK* study provided us with information on the participants' SES, but their average number of contacts is available only in the aggregate form. Thus, from the data, we could compute only $G_{i\alpha,j}$ which is the average number of contacts that an individual of age group $i$ and SES $\alpha$ has with all the individuals of age group $j$. To disaggregate the average number of contacts they had with each SES group we introduced some assumptions. Namely, we assumed assortative mixing along SES. We assigned the average number of contactees to the different SES as follows: $G_{i\alpha,j\beta} = G_{i\alpha,j} u_{\alpha\beta}$ where $u_{\alpha\beta}$ is a parameter that controls how these contacts are distributed among individuals of different SES. To mimic assortativity patterns among SES, the values of $u_{\alpha\beta}$ are set as follows:

$$u_{\alpha\beta} = \begin{pmatrix} 0.7 & 0.2 & 0.1 \\ 0.1 & 0.7 & 0.2 \\ 0.1 & 0.2 & 0.7 \end{pmatrix} \tag{S44}$$

We note higher values in the diagonal, meaning that 70% of children's contacts are with children belonging to the same SES. The average number of contacts of these matrices includes the contacts of the family members. However, contacts have been added afterwards to the matrix by assuming that individuals belonging to the same family have the same SES.

## 7 Impact of generalized contact matrices on epidemic modeling

To provide evidence of the robustness of the proposed approach we analyze contact data from a second country. In particular, we consider the age-structured contact matrix $\mathbf{C}$ of Zimbabwe [60] (Fig. S2-a). We note how $\mathbf{C}$ is different from the Hungarian one. Indeed, although the age assortative and the inter-generational interaction are still clear, the matrix shows a high activity rate for individuals aged 80+. By applying the same analysis that we did for the Hungarian data we study the impact of generalized

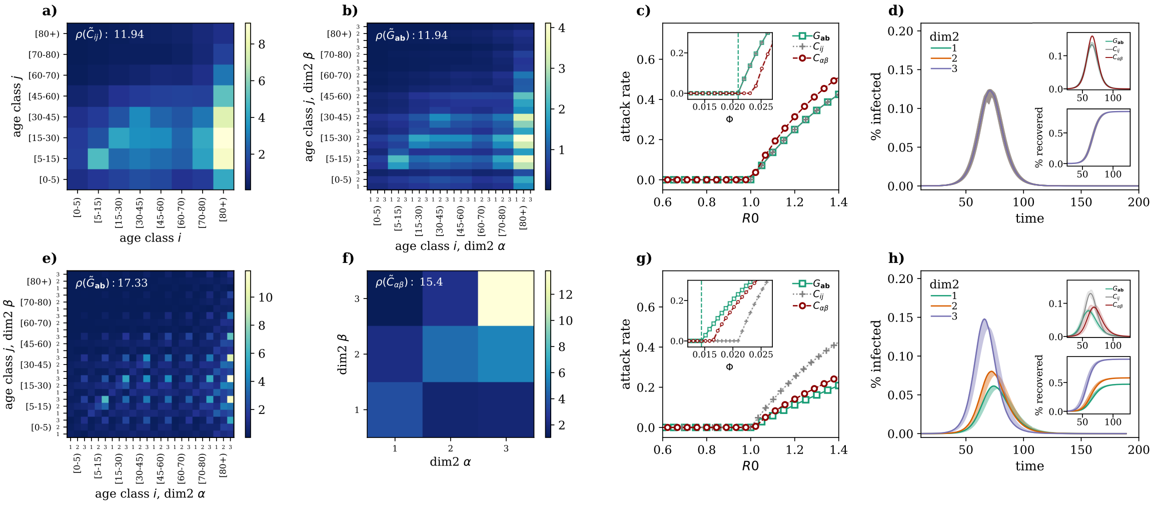


Fig. S2: **Impact of generalized contact matrices on epidemic spreading**. (a) Example of a classic age contact matrix $C_{ij}$ $(8 \times 8)$; (b) and (e) generalized contact matrices with an additional dimension with three levels $G_{\mathbf{ab}}$ $(24 \times 24)$ in the case of random mixing among the second level groups (b) and in the case of assortative mixing and different activity levels among the second dimension (e); (f) depicts the case when we integrate the generalized contact matrix over all age classes; (c) attack rate as a function of $R_0$ (main figure) and disease transmissibility (inset). In panel (d) we show, in the case of random mixing, the prevalence (main figure and top inset) and the fraction of recovered as a function of time (bottom inset); (g) and (h) are as the previous two plots but for the case of assortative mixing with different levels of activity. Results refer to the median of 500 runs. Epidemiological parameters: $\Gamma = 0.25$, $\Psi = 0.4$, and $R_0 = 2.7$. Simulations start with a number $I_0 = 100$ of initial infectious seeds.

contact matrices on epidemic modeling in $(i)$ the random mixing (Fig. S2-b) and, $(ii)$ the assortative mixing (Fig. S2-e) regimes. As in the main text, we imagine a simple case where the second dimension contains three groups: $1, 2, 3$. We assume that 35%, 45%, and 20% of the population belong to these three categories across all age groups. For the assortative mixing scenario, we assume that 60%, 50%, and 65% of the contacts in the first, second, and third groups of the additional category take place within each group. Additionally, we assume some levels of heterogeneity also in the activity of the different groups, setting to 20%, 40%, and 40% the share of contacts of the three groups. In Fig. S2-f we show the matrix $C_{\alpha\beta}$ obtained by integrating the latter generalized contact matrix over all age classes.

We use these matrices to study the unfolding of a virus in the population. In Fig. S2-c we plot the attack rate (i.e., epidemic size) as a function of $R_0$ and as a function of the transmissibility parameter $\Phi$ (inset) for 1) a model fed only with the contact matrix $C_{ij}$ (grey crosses), 2) a model fed with the generalized contact matrix $G_{\mathbf{a},\mathbf{b}}$ (green squares), 3) a model fed with the matrix $C_{\alpha\beta}$ where contacts are stratified only according to the second dimension (red circles). In Fig. S2-d we show the prevalence of infected overtime of the three dimensions as predicted by the model fed with $G_{\mathbf{a},\mathbf{b}}$ for the three groups. In the top inset, we show the the overall prevalence of infected predicted by the three models. In the bottom inset, we plot the fraction of recovered as a function of time for the three groups in the second dimension. We perform the same analysis for the assortative scenario and we show the results respectively in Fig. S2-g and Fig. S2-h. All the results are in perfect agreement with the ones discussed in the main text.

# 8 Sensitivity analysis

In this section, we perform a sensitivity analysis of the model to build synthetic generalized contact matrices. In particular, we show how the spectral radius of $G_{\mathbf{ab}}$ differs from the one computed on the aggregate age contact $C_{ij}$ when we vary some of the parameters of the model.

We create nine different scenarios one for each combination of three sets of values for the population distribution of the second dimension and three sets of parameters for the assortativity. For each of these scenarios in Fig. S3 we show the ratio of the $\rho(\tilde{G}_{\mathbf{ab}})$ over $\rho(\tilde{C}_{ij})$ as function of the activity of individuals in dim1 $(P_1)$ and, of individuals in dim2 $(P_2)$. Given that $\sum_1^3 P_i = 1$ for each $P_1$ and $P_2$, $P_3 = 1 - P_1 - P_2$,

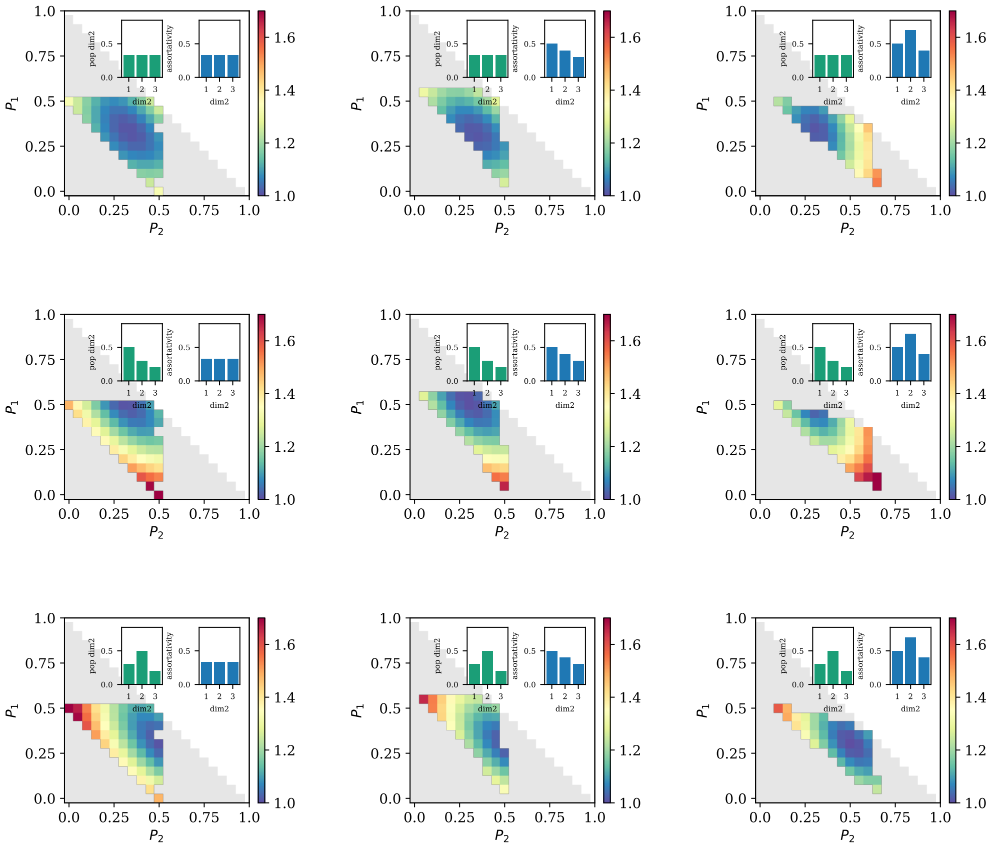


Fig. S3: **Sensitivity analysis**. Variation of the ratio of the $\rho(G_{\mathbf{ab}})$ over $\rho(C_{ij})$ as a function of the activities $P_1, P_2$. Given that $\sum_1^3 P_i = 1$ for each $P_1$ and $P_2$, $P_3 = 1 - P_1 - P_2$. In each subplot $G_{\mathbf{ab}}$ is computed according to our synthetic model using different parameters distribution of population distribution of the second dimension (left inset) and assortativity along the second dimension (right inset). The grey area refers to the non-physical solutions of the model.

thus each pair of values of $P_1$, $P_2$ defines also the value of $P_3$.

As shown above, $\rho(\tilde{G}_{\mathbf{ab}})$ is always grater or equal to $\rho(\tilde{C}_{ij})$ as expected from the theory. From Fig. S3 we can observe how the parameter sets of the different scenarios influence the magnitude of this relation, suggesting that different interaction patterns arising from correlation to an additional dimension effectively impact the estimation of the basic reproductive number.

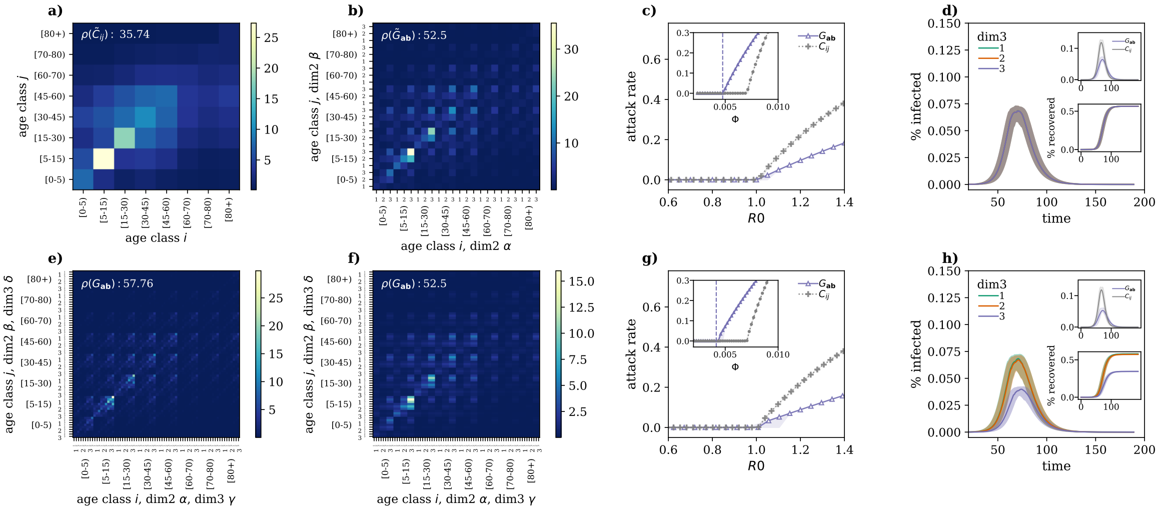

Fig. S4: **Three dimensions generalized contact matrices**. (a) Example of a classic age contact matrix $C_{ij}$ $(8 \times 8)$; (b) Generalized contact matrices with an additional dimension with two levels $G_{\mathbf{ab}}$ $(24 \times 24)$ in the case of assortative mixing and different activity levels among the second dimension (e) and (f) Generalized contact matrices with three levels $G_{\mathbf{ab}}$ $(72 \times 72)$ (e) in the case of assortative mixing and different activity levels among the third dimension and (f) in the case of random mixing among the third dimension (e); (c) attack rate as a function of $R_0$ (main figure) and disease transmissibility (inset). In panel (d) we show, in the case of random mixing, the prevalence (main figure and top inset) and the fraction of recovered as function of time (bottom inset); (g) and (h) are as the previous two plots but for the case of assortative mixing with different levels of activity. Results refer to the median of 500 runs. Epidemiological parameters: $\Gamma = 0.25, \Psi = 0.4$, and $R_0 = 2.7$. Simulations start with a number $I_0 = 100$ of initial infectious seeds.

## 8.1 Testing the model with three dimensions

To show the validity of our approach beyond the two dimensions here we present the results of our analysis when we stratify the matrix along three dimensions (age, dim2, and dim3). We assume that also the third dimension has three possible groups. Starting from a generalized contact matrix with two dimensions (Fig. S4-b) we build a generalized contact matrix with the assumption of random mixing among the third dimension (Fig. S4-f) and assortative assumption along the third dimension (Fig. S4-e). We assume the same parameters as for the assortative regime of Fig. 2 in the main text to build the 2D matrix. For the third dimension, we assume that individuals are respectively 30%, 30% and 40% in the three dimensions. Additionally, we assume that 34%, 33% and 46% represent the respective proportion of assortative contacts for the three groups and that 40%, 40% and 20% indicate the relative activity of the three groups. Also in this case, we use these matrices to study the unfolding of a virus in the population. We can observe that, in the assortative mixing scenario, the spectral radius of the $G_{\mathbf{a},\mathbf{b}}$ with three dimensions (Fig. S4-e) is higher than the one of $G_{\mathbf{a},\mathbf{b}}$ with two dimensions (Fig. S4-b). This implies that, given $R_0 = 1$, the critical value of $\Phi$ predicted by the model with three dimensions is going to be smaller than the one predicted by the model with two dimensions.

In Fig. S4-c we plot the attack rate (i.e., epidemic size) as a function of $R_0$ and as a function of the transmissibility parameter $\Phi$ (inset) for 1) a model fed only with the contact matrix $C_{ij}$ (grey crosses), 2) a model fed with the generalized contact matrix $G_{\mathbf{a},\mathbf{b}}$ with three dimensions (purple triangles). In Fig S4-d we show the prevalence of infected overtime of the three dimensions as predicted by the model fed with $G_{\mathbf{a},\mathbf{b}}$ with three dimensions for the three groups of *dim*3. In the top inset, we show the overall prevalence of infection predicted by the two models. In the bottom inset instead, we plot the fraction of recovered as a function of time for the groups in the third dimension. We perform the same analysis for the assortative scenario and we show the results respectively in Fig S4-g and Fig S4-h. All the results are in perfect agreement with the ones discussed in the main text.

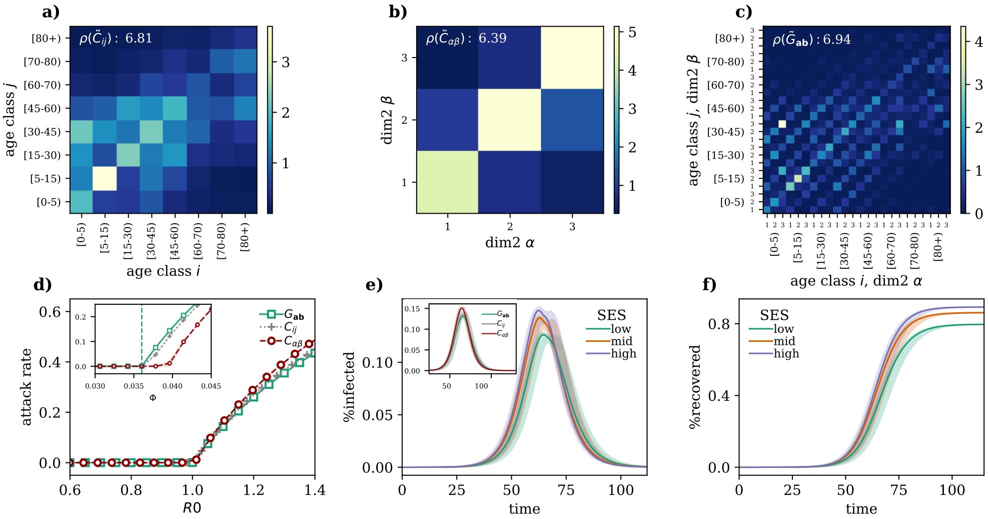


Fig. S5: **Empirical generalized contact matrices.** (a) Age contact matrix $C_{ij}$ ($8 \times 8$); (b) Socio-economic status (SES) contact matrix $C_{\alpha\beta}$ ($3 \times 3$) and (c) Generalized contact matrix with age and socio-economic status $G_{\mathbf{ab}}$ ($24 \times 24$); (d) attack rate as a function of $R_0$, (e) disease prevalence as a function of time, and (f) fraction of recovered as function of time. Results refer to the median of 500 runs. Epidemiological parameters: $\mu = 0.25, \epsilon = 0.4$, $R_0 = 2.7$, $I_0 = 100$. The matrices have been computed using the contact diaries coming from the MASZK data collected in Hungary during April 2021.

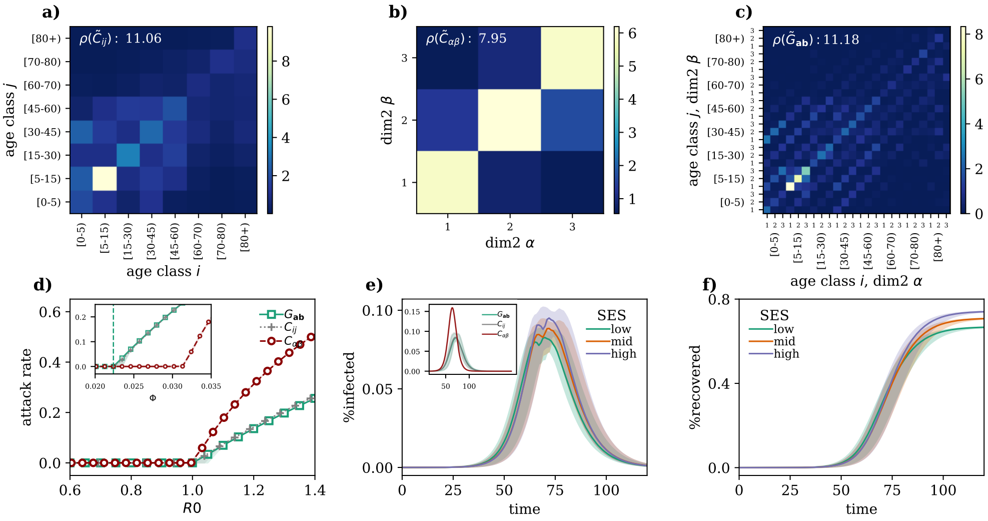


Fig. S6: **Empirical generalized contact matrices.** (a) Age contact matrix $C_{ij}$ ($8 \times 8$); (b) Socio-economic status (SES) contact matrix $C_{\alpha\beta}$ ($3 \times 3$) and (c) Generalized contact matrix with age and socio-economic status $G_{\mathbf{ab}}$ ($24 \times 24$); (d) attack rate as a function of $R_0$ (e) disease prevalence as a function of time and (f) fraction of recovered as function of time. Results refer to the median of 500 runs. Epidemiological parameters: $\mu = 0.25, \epsilon = 0.4$, $R_0 = 2.7$, $I_0 = 100$. The matrices have been computed using the contact diaries from the MASZK data collected in Hungary during November 2021.

## 8.2 Empirical generalized contact data

The MASZK questionnaire provided us with three diaries that we were able to use to generate the generalized contact matrices stratifying by age and SES of participants. Here, we show the results of the same analysis shown in the main text for two additional periods: April (Fig. S5) and November (Fig. S6) 2021. While the overall results confirm the one presented in the main text, models fed with standard and generalized contact matrices lead to estimations of the attack rates, for a given $R_0$, which are closer to each other. Nevertheless, the model featuring generalized contact matrices allows capturing heterogeneities in the incidence across SES groups which are invisible to standard approaches.

## REFERENCES AND NOTES

1. W. J. Edmunds, C. J. O'callaghan, D. J. Nokes, Who mixes with whom? A method to determine the contact patterns of adults that may lead to the spread of airborne infections. *Proc. Biol. Sci.* **264**, 949–957 (1997).

2. J. Mossong, N. Hens, M. Jit, P. Beutels, K. Auranen, R. Mikolajczyk, M. Massari, S. Salmaso, G. S. Tomba, J. Wallinga, J. Heijne, M. Sadkowska-Todys, M. Rosinska, W. J. Edmunds, Social contacts and mixing patterns relevant to the spread of infectious diseases. *PLoS Med.* **5**, e74 (2008).

3. K. Prem, A. R. Cook, M. Jit, Projecting social contact matrices in 152 countries using contact surveys and demographic data. *PLoS Comput. Biol.* **13**, e1005697 (2017).

4. P. Rohani, X. Zhong, A. A. King, Contact network structure explains the changing epidemiology of pertussis. *Science* **330**, 982–985 (2010).

5. D. Mistry, M. Litvinova, A. Pastore, Y. Piontti, M. Chinazzi, L. Fumanelli, M. F. C. Gomes, S. A. Haque, Q.-H. Liu, K. Mu, X. Xiong, M. E. Halloran, I. M. Longini, S. Merler, M. Ajelli, A. Vespignani, Inferring high-resolution human mixing patterns for disease modeling. *Nat. Commun.* **12**, 323 (2021).

6. J. Wallinga, P. Teunis, M. Kretzschmar, Using data on social contacts to estimate age-specific transmission parameters for respiratory-spread infectious agents. *Am. J. Epidemiol.* **164**, 936–944 (2006).

7. T. Hoang, P. Coletti, A. Melegaro, J. Wallinga, C. G. Grijalva, J. W. Edmunds, P. Beutels, N. Hens, A systematic review of social contact surveys to inform transmission models of close-contact infections. *Epidemiology* **30**, 723 (2019).

8. L. D. Amico, J. Kleynhans, L. Gauvin, M. Tizzoni, L. Ozella, M. Makhasi, N. Wolter, B. Language, R. G. Wagner, C. Cohen, S. Tempia, C. Cattuto, Estimating household contact matrices structure from easily collectable metadata. *PLOS ONE* **19**, 1–13 (2024).

9. R. M. Anderson, R. M. May, Age-related changes in the rate of disease transmission: Implications for the design of vaccination programmes. *Epidemiol. Infect.* **94**, 365–436 (1985).

10. R. M. Anderson, R. M. May, Vaccination against rubella and measles: Quantitative investigations of different policies. *Epidemiol. Infect.* **90**, 259–325 (1983).

11. R. Verity, L. C. Okell, I. Dorigatti, P. Winskill, C. Whittaker, N. Imai, G. Cuomo-Dannenburg, H. Thompson, P. G. T. Walker, F. Han, A. Dighe, J. T. Griffin, M. Baguelin, S. Bhatia, A. Boonyasiri, A. Cori, Z. Cucunubá, R. FitzJohn, K. Gaythorpe, W. Green, A. Hamlet, W. Hinsley, D. Laydon, G. Nedjati-Gilani, S. Riley, S. van Elsland, E. Volz, H. Wang, Y. Wang, X. Xi, C. A. Donnelly, A. C. Ghani, N. M. Ferguson, Estimates of the severity of coronavirus disease 2019: A model-based analysis. *Lancet Infect. Dis.* **20**, 669–677 (2020).

12. L. Fumanelli, M. Ajelli, P. Manfredi, A. Vespignani, S. Merler, Inferring the structure of social contacts from demographic data in the analysis of infectious diseases spread. *PLoS Comput. Biol.* **8**, e1002673 (2012).

13. N. Hens, G. M. Ayele, N. Goeyvaerts, M. Aerts, J. Mossong, J. W. Edmunds, P. Beutels, Estimating the impact of school closure on social mixing behaviour and the transmission of close contact infections in eight European countries. *BMC Infect. Dis.* **9**, 1–12 (2009).

14. N. Goeyvaerts, N. Hens, B. Ogunjimi, M. Aerts, Z. Shkedy, P. Van Damme, P. Beutels, Estimating infectious disease parameters from data on social contacts and serological status. *J. R. Stat. Soc. Ser. C Appl. Stat.* **59**, 255–277 (2010).

15. N. Goeyvaerts, E. Santermans, G. Potter, A. Torneri, K. Van Kerckhove, L. Willem, M. Aerts, P. Beutels, N. Hens, Household members do not contact each other at random: Implications for infectious disease modelling. *Proc. R. Soc. B* **285**, 20182201 (2018).

16. A. Aleta, D. Martín-Corral, M. A. Bakker, A. Pastore, Y. Piontti, M. Ajelli, M. Litvinova, M. Chinazzi, N. E. Dean, M. E. Halloran, I. M. Longini Jr., A. Pentland, A. Vespignani, Y. Moreno, E. Moro, Quantifying the importance and location of SARS-CoV-2 transmission events in large metropolitan areas. *Proc. Natl. Acad. Sci. U.S.A.* **119**, e2112182119 (2022).

17. C. Buckee, A. Noor, L. Sattenspiel, Thinking clearly about social aspects of infectious disease transmission. *Nature* **595**, 205–213 (2021).

18. M. Tizzoni, E. O. Nsoesie, L. Gauvin, M. Karsai, N. Perra, S. Bansal, Addressing the socioeconomic divide in computational modeling for infectious diseases. *Nat. Commun.* **13**, 1–7 (2022).

19. J. Bedson, L. A. Skrip, D. Pedi, S. Abramowitz, S. Carter, M. F. Jalloh, S. Funk, N. Gobat, T. Giles-Vernick, G. Chowell, J. Rangel, J. R. de Almeida, R. Elessawi, S. V. Scarpino, R. A. Hammond, S. Briand, J. M. Epstein, L. Hébert-Dufresne, B. M. Althouse, A review and agenda for integrated disease models including social and behavioural factors. *Nat. Hum. Behav.* **5**, 834–846 (2021).

20. J. Zelner, N. B. Masters, R. Naraharisetti, S. A. Mojola, M. Chowkwanyun, R. Malosh, There are no equal opportunity infectors: Epidemiological modelers must rethink our approach to inequality in infection risk. *PLoS Comput. Biol.* **18**, e1009795 (2022).

21. K. H. Grantz, M. S. Rane, H. Salje, G. E. Glass, S. E. Schachterle, D. A. Cummings, Disparities in influenza mortality and transmission related to sociodemographic factors within Chicago in the pandemic of 1918. *Proc. Natl. Acad. Sci. U.S.A.* **113**, 13839–13844 (2016).

22. S.-E. Mamelund, C. Shelley-Egan, O. Rogeberg, The association between socioeconomic status and pandemic influenza: Systematic review and meta-analysis. *PLOS ONE* **16**, e0244346 (2021).

23. K. A. Alexander, C. E. Sanderson, M. Marathe, B. L. Lewis, C. M. Rivers, J. Shaman, J. M. Drake, E. Lofgren, V. M. Dato, M. C. Eisenberg, S. Eubank, What factors might have led to the emergence of Ebola in West Africa? *PLoS Negl. Trop. Dis.* **9**, e0003652 (2015).

24. N. Perra, Non-pharmaceutical interventions during the COVID-19 pandemic: A review. *Phys. Rep.* **913**, 1–52 (2021).

25. R. Garnier, J. R. Benetka, J. Kraemer, S. Bansal, Socioeconomic disparities in social distancing during the COVID-19 pandemic in the United States: Observational study. *J. Med. Internet Res.* **23**, e24591 (2021).

26. W. Do Lee, M. Qian, T. Schwanen, The association between socioeconomic status and mobility reductions in the early stage of England's COVID-19 epidemic. *Health Place* **69**, 102563 (2021).

27. N. Gozzi, M. Tizzoni, M. Chinazzi, L. Ferres, A. Vespignani, N. Perra, Estimating the effect of social inequalities on the mitigation of COVID-19 across communities in Santiago de Chile. *Nat. Commun.* **12**, 1–9 (2021).

28. S. Heroy, I. Loaiza, A. Pentland, N. O'Clery, COVID-19 policy analysis: Labour structure dictates lockdown mobility behaviour. *J. R. Soc. Interface* **18**, 20201035 (2021).

29. L. Gauvin, P. Bajardi, E. Pepe, B. Lake, F. Privitera, M. Tizzoni, Socio-economic determinants of mobility responses during the first wave of COVID-19 in Italy: From provinces to neighbourhoods. *J. R. Soc. Interface* **18**, 20210092 (2021).

30. J. A. Weill, M. Stigler, O. Deschenes, M. R. Springborn, Social distancing responses to COVID-19 emergency declarations strongly differentiated by income. *Proc. Natl. Acad. Sci. U.S.A.* **117**, 19658–19660 (2020).

31. J. Jay, J. Bor, E. O. Nsoesie, S. K. Lipson, D. K. Jones, S. Galea, J. Raifman, Neighbourhood income and physical distancing during the COVID-19 pandemic in the United States. *Nat. Hum. Behav.* **4**, 1294–1302 (2020).

32. G. Bonaccorsi, F. Pierri, M. Cinelli, A. Flori, A. Galeazzi, F. Porcelli, A. L. Schmidt, C. M. Valensise, A. Scala, W. Quattrociocchi, F. Pammolli, Economic and social consequences of human mobility restrictions under COVID-19. *Proc. Natl. Acad. Sci. U.S.A.* **117**, 15530–15535 (2020).

33. S. J. Fox, E. Javan, R. Pasco, G. C. Gibson, B. Betke, J. L. Herrera-Diestra, S. Woody, K. Pierce, K. E. Johnson, M. Johnson-León, M. Lachmann, L. A. Meyers, Disproportionate impacts of COVID-19 in a large US city. *PLoS Comput. Biol.* **19**, e1011149 (2023).

34. N. Gozzi, M. Chinazzi, N. E. Dean, I. M. Longini Jr, M. Elizabeth Halloran, N. Perra, A. Vespignani, Estimating the impact of COVID-19 vaccine inequities: A modeling study. *Nat. Commun.* **14**, 3272 (2023).

35. A. Mousa, P. Winskill, O. J. Watson, O. Ratmann, M. Monod, M. Ajelli, A. Diallo, P. J. Dodd, C. G. Grijalva, M. C. Kiti, A. Krishnan, R. Kumar, S. Kumar, K. O. Kwok, C. F. Lanata, A. Melegaro, C. D. Morrow, J. Mossong, E. F. G. Neal, D. J. Nokes, W. Pan-ngum, G. E. Potter, F. M. Russell, S. Saha, J. D. Sugimoto, W. In Wei, R. R. Wood, J. T. Wu, J. Zhang, P. G. T. Walker, C. Whittaker, Social contact patterns and implications for infectious disease transmission–A systematic review and meta-analysis of contact surveys. *eLife* **10**, e70294 (2021).

36. O. Diekmann, J. A. Heesterbeek, M. G. Roberts, The construction of next-generation matrices for compartmental epidemic models. *J. R. Soc. Interface* **7**, 873–885 (2010).

37. C. B. Julie, L. M. Childs, An introduction to compartmental modeling for the budding infectious disease modeler. *Lett. Biomath.* **5**, 195–221 (2010).

38. P. Rohani M. Keeling, *Modeling Infectious Diseases in Humans and Animals* (Princeton Univ. Press, 2008).

39. A. Barrat, M. Barthelemy, A. Vespignani, *Dynamical Processes on Complex Networks* (Cambridge Univ. Press, 2012).

40. J. Koltai, O. Vásárhelyi, G. Röst, M. Karsai, Reconstructing social mixing patterns via weighted contact matrices from online and representative surveys. *Sci. Rep.* **12**, 1–12 (2022).

41. K. Sun, A. Baronchelli, N. Perra, Contrasting effects of strong ties on SIR and SIS processes in temporal networks. *Eur. Phys. J. B.* **88**, 1–8 (2015).

42. L. Di Domenico, G. Pullano, C. E. Sabbatini, P.-Y. Boëlle, V. Colizza, Impact of lockdown on COVID-19 epidemic in Île-de-France and possible exit strategies. *BMC Med.* **18**, 1–13 (2020).

43. Y. Leo, E. Fleury, J. I. Alvarez-Hamelin, C. Sarraute, M. Karsai, Socioeconomic correlations and stratification in social-communication networks. *J. R. Soc. Interface* **13**, 20160598 (2016).

44. X. Dong, A. J. Morales, E. Jahani, E. Moro, B. Lepri, B. Bozkaya, C. Sarraute, Y. Bar-Yam, A. Pentland, Segregated interactions in urban and online space. *EPJ Data Sci.* **9**, 7 (2020).

45. B. Oroszi, A. Juhász, C. Nagy, J. K. Horváth, K. E. Komlós, G. Túri, M. McKee, R. Ádány, Characteristics of the third COVID-19 pandemic wave with special focus on socioeconomic inequalities in morbidity, mortality and the uptake of COVID-19 vaccination in Hungary. *J. Pers. Med.* **12**, 388 (2022).

46. E. Valdano, J. Lee, S. Bansal, S. Rubrichi, V. Colizza, Highlighting socio-economic constraints on mobility reductions during COVID-19 restrictions in France can inform effective and equitable pandemic response. *J. Travel Med.* **28**, taab045 (2021).

47. M. Karmakar, P. M. Lantz, R. Tipirneni, Association of social and demographic factors with COVID-19 incidence and death rates in the US. *JAMA Netw. Open* **4**, e2036462 (2021).

48. J. Zelner, R. Trangucci, R. Naraharisetti, A. Cao, R. Malosh, K. Broen, N. Masters, P. Delamater, Racial disparities in coronavirus disease 2019 (COVID-19) mortality are driven by unequal infection risks. *Clin. Infect. Dis.* **72**, e88–e95 (2021).

49. J. Y.-M. Siu, Health inequality experienced by the socially disadvantaged populations during the outbreak of COVID-19 in Hong Kong: An interaction with social inequality. *Health Soc. Care Community* **29**, 1522–1529 (2021).

50. S. Sweeney, T. P. J. Capeding, R. Eggo, M. Huda, M. Jit, D. Mudzengi, N. R. Naylor, S. Procter, M. Quaife, L. Serebryakova, S. Torres-Rueda, V. Vargas, CHiL COVID Working

Group, A. Vassall, Exploring equity in health and poverty impacts of control measures for SARS-CoV-2 in six countries. *BMJ Glob. Health* **6**, e005521 (2021).

51. K. C. Ma, T. F. Menkir, S. Kissler, Y. H. Grad, M. Lipsitch, Modeling the impact of racial and ethnic disparities on COVID-19 epidemic dynamics. *eLife* **10**, e66601 (2021).

52. S. Chang, E. Pierson, P. W. Koh, J. Gerardin, B. Redbird, D. Grusky, J. Leskovec, Mobility network models of COVID-19 explain inequities and inform reopening. *Nature* **589**, 82–87 (2021).

53. A. Aleta, D. Martin-Corral, A. Pastore, Y. Piontti, M. Ajelli, M. Litvinova, M. Chinazzi, N. E. Dean, M. E. Halloran, I. M. Longini Jr., S. Merler, A. Pentland, A. Vespignani, E. Moro, Y. Moreno, Modelling the impact of testing, contact tracing and household quarantine on second waves of COVID-19. *Nat. Hum. Behav.* **4**, 964–971 (2020).

54. M. Pangallo, A. Aleta, R. M. D. Rio-Chanona, A. Pichler, D. Martín-Corral, M. Chinazzi, F. Lafond, M. Ajelli, E. Moro, Y. Moreno, A. Vespignani, J. Doyne, Farmer, The unequal effects of the health–economy trade-off during the covid-19 pandemic. *Nat. Hum. Behav.* **8**, 264–275 (2024).

55. A. Machens, F. Gesualdo, C. Rizzo, A. E. Tozzi, A. Barrat, C. Cattuto, An infectious disease model on empirical networks of human contact: Bridging the gap between dynamic network data and contact matrices. *BMC Infect. Dis.* **13**, 185 (2013).

56. A. Aleta, G. F. de Arruda, Y. Moreno, Data-driven contact structures: From homogeneous mixing to multilayer networks. *PLoS Comput. Biol.* **16**, e1008035 (2020).

57. J. C. Miller, E. M. Volz, Incorporating disease and population structure into models of SIR disease in contact networks. *PLOS ONE* **8**, e69162 (2013).

58. M. Ajelli, B. Gonçalves, D. Balcan, V. Colizza, H. Hao, J. J. Ramasco, S. Merler, A. Vespignani, Comparing large-scale computational approaches to epidemic modeling: Agent-based versus structured metapopulation models. *BMC Infect. Dis.* **10**, 190 (2010).

59. P. Klepac, S. Kissler, J. Gog, Contagion! the BBC four pandemic–The model behind the documentary. *Epidemics* **24**, 49–59 (2018).

60. A. Melegaro, E. Del Fava, P. Poletti, S. Merler, C. Nyamukapa, J. Williams, S. Gregson, P. Manfredi, Social contact structures and time use patterns in the Manicaland Province of Zimbabwe. *PLOS ONE* **12**, e0170459 (2017).

61. M. Ajelli, M. Litvinova, Estimating contact patterns relevant to the spread of infectious diseases in Russia. *J. Theor. Biol.* **419**, 1–7 (2017).

62. J. Zhang, M. Litvinova, Y. Liang, Y. Wang, W. Wang, S. Zhao, W. Qianhui, S. Merler, C. Viboud, A. Vespignani, M. Ajelli, Y. Hongjie, Changes in contact patterns shape the dynamics of the COVID-19 outbreak in China. *Science* **368**, 1481–1486 (2020).

63. A. Gimma, J. D. Munday, K. L. M. Wong, P. Coletti, K. van Zandvoort, K. Prem, P. Klepac, G. J. Rubin, S. Funk, W. J. Edmunds, C. I. Jarvis, CMMID COVID-19 working group, Changes in social contacts in England during the COVID-19 pandemic between March 2020 and March 2021 as measured by the CoMix survey: A repeated cross-sectional study. *PLoS Med.* **19**, e1003907 (2022).

64. E. Moro, D. Calacci, X. Dong, A. Pentland, Mobility patterns are associated with experienced income segregation in large US cities. *Nat. Commun.* **12**, 4633 (2021).

65. T. Yabe, B. G. B. Bueno, X. Dong, A. Pentland, E. Moro, Behavioral changes during the COVID-19 pandemic decreased income diversity of urban encounters. *Nat. Commun.* **14**, 2310 (2023).

66. L. Rocher, J. M. Hendrickx, Y.-A. De Montjoye, Estimating the success of re-identifications in incomplete datasets using generative models. *Nat. Commun.* **10**, 3069 (2019).

67. N. Oliver, B. Lepri, H. Sterly, R. Lambiotte, S. Deletaille, M. De Nadai, E. Letouzé, A. A. Salah, R. Benjamins, C. Cattuto, V. Colizza, N. de Cordes, S. P. Fraiberger, T. Koebe, S. Lehmann, J. Murillo, A. Pentland, P. N. Pham, F. Pivetta, J. Saramäki, S. V. Scarpino, M.

Tizzoni, S. Verhulst, P. Vinck, Mobile phone data for informing public health actions across the COVID-19 pandemic life cycle. *Sci. Adv.* **6**, eabc0764 (2020).

68. M. Karsai, J. Koltai, O. Vásárhelyi, G. Röst, Hungary in mask/maszk in Hungary. *Corvinus J. Sociol. Soc. Policy* **2**, 139–146 (2020).

69. C. D. Meyer, *Matrix Analysis and Applied Linear Algebra* (SIAM, 2023).

70. R. A. Horn, C. R. Johnson, *Matrix Analysis* (Cambridge Univ. Press, 2012.

71. Hungarian National Authority for Data Protection and Freedom of Information, *Nemzeti adatvédelmi és információ szabadság hatóság,* https://www.naih.hu/, 2023.

72. European Centre for Disease Prevention and Control, Surveillance definitions for COVID-19; www.ecdc.europa.eu/en/covid-19/surveillance/surveillance-definitions.

73. S. Arregui, A. Aleta, J. Sanz, Y. Moreno, Projecting social contact matrices to different demographic structures. *PLoS Comput. Biol.* **14**, e1006638 (2018).
